# Genome sequence of the progenitor of the wheat D genome *Aegilops tauschii*

**DOI:** 10.1038/nature24486

**Published:** 2017-11-01

**Authors:** Ming-Cheng Luo, Yong Q. Gu, Daniela Puiu, Hao Wang, Sven O. Twardziok, Karin R. Deal, Naxin Huo, Tingting Zhu, Le Wang, Yi Wang, Patrick E. McGuire, Shuyang Liu, Hai Long, Ramesh K. Ramasamy, Juan C. Rodriguez, Sonny L. Van, Luxia Yuan, Zhenzhong Wang, Zhiqiang Xia, Lichan Xiao, Olin D. Anderson, Shuhong Ouyang, Yong Liang, Aleksey V. Zimin, Geo Pertea, Peng Qi, Jeffrey L. Bennetzen, Xiongtao Dai, Matthew W. Dawson, Hans-Georg Müller, Karl Kugler, Lorena Rivarola-Duarte, Manuel Spannagl, Klaus F. X. Mayer, Fu-Hao Lu, Michael W. Bevan, Philippe Leroy, Pingchuan Li, Frank M. You, Qixin Sun, Zhiyong Liu, Eric Lyons, Thomas Wicker, Steven L. Salzberg, Katrien M. Devos, Jan Dvořák

**Affiliations:** 1 Department of Plant Sciences, University of California, Davis, California USA; 2 Crop Improvement & Genetics Research, USDA-ARS, Albany, California USA; 3 Center for Computational Biology, McKusick-Nathans Institute of Genetic Medicine, Johns Hopkins University School of Medicine, Baltimore, Maryland USA; 4 Department of Crop & Soil Sciences, Institute of Plant Breeding, Genetics and Genomics, University of Georgia, Athens, Georgia USA; 5 Department of Plant Biology, University of Georgia, Athens, Georgia USA; 6 Department of Genetics, University of Georgia, Athens, Georgia USA; 7 Plant Genome and Systems Biology, Helmholtz Zentrum München, Neuherberg, Germany; 8 China Agricultural University, Beijing, China; 9 Department of Statistics, University of California, Davis, California USA; 10 School of Life Sciences Weihenstephan, Technical University of Munich, Munich, Germany; 11 Department Cell and Developmental Biology, John Innes Centre, Norwich Research Park, Norwich, UK; 12 INRA, UBP, UMR 1095, GDEC, Clermont-Ferrand France; 13 Agriculture & Agri-Food Canada, Morden, Winnipeg Canada; 14 CyVerse, University of Arizona, Tucson, Arizona USA; 15 Department of Plant and Microbial Biology, University of Zürich, Zürich, Switzerland; 16 Departments of Biomedical Engineering, Computer Science, and Biostatistics, Johns Hopkins University, Baltimore, Maryland USA

**Keywords:** Genome evolution, Plant evolution

## Abstract

*Aegilops tauschii* is the diploid progenitor of the D genome of hexaploid wheat^1^ (*Triticum aestivum*, genomes AABBDD) and an important genetic resource for wheat^2,3,4^. The large size and highly repetitive nature of the *Ae. tauschii* genome has until now precluded the development of a reference-quality genome sequence^5^. Here we use an array of advanced technologies, including ordered-clone genome sequencing, whole-genome shotgun sequencing, and BioNano optical genome mapping, to generate a reference-quality genome sequence for *Ae. tauschii* ssp. *strangulata* accession AL8/78, which is closely related to the wheat D genome. We show that compared to other sequenced plant genomes, including a much larger conifer genome, the *Ae. tauschii* genome contains unprecedented amounts of very similar repeated sequences. Our genome comparisons reveal that the *Ae. tauschii* genome has a greater number of dispersed duplicated genes than other sequenced genomes and its chromosomes have been structurally evolving an order of magnitude faster than those of other grass genomes. The decay of colinearity with other grass genomes correlates with recombination rates along chromosomes. We propose that the vast amounts of very similar repeated sequences cause frequent errors in recombination and lead to gene duplications and structural chromosome changes that drive fast genome evolution.

**Supplementary information:**

The online version of this article (doi:10.1038/nature24486) contains supplementary material, which is available to authorized users.

## Abstract (web summary)

A combination of advanced sequencing and mapping techniques is used to produce a reference genome of *Aegilops tauschii*, progenitor of the wheat D genome, providing a valuable resource for comparative genetic studies.

**Supplementary information:**

The online version of this article (doi:10.1038/nature24486) contains supplementary material, which is available to authorized users.

## Genome of common wheat ancestor

Sequencing the genomes of crops plants provides useful resources for crop improvement and breeding. Jan Dvořák, Katrien Devos, Steven Salzberg and colleagues report a reference genome for *Aegilops tauschii*, the diploid progenitor of the D genome of hexaploid wheat. They use a combination of ordered-clone genome sequencing, whole-genome shotgun sequencing and BioNano optical genome mapping to assemble this large and highly repetitive genome. This provides a useful resource for comparative genomics studies of wheat.

**Supplementary information:**

The online version of this article (doi:10.1038/nature24486) contains supplementary material, which is available to authorized users.

## Main

**Extended Data Figure 1:**
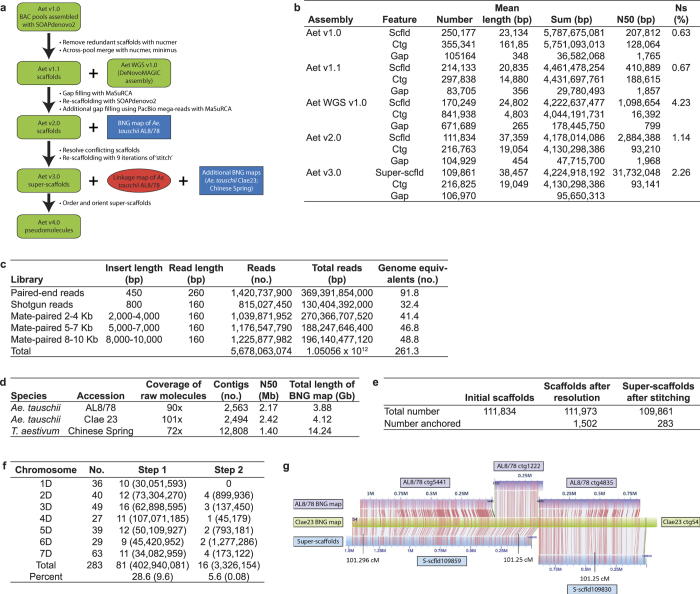
Scaffold and super-scaffold assembly. **a**, Flowchart of the scaffold and pseudomolecule assembly. **b**, Numbers and lengths of contigs (ctg), scaffolds (scfld), super-scaffolds (super-scfld), and gaps in the sequential *Ae. tauschii* genome assemblies culminating with assembly Aet v3.0. **c**, *Ae. tauschii* WGS libraries sequenced with Illumina HiSeq 2500. **d**, The development of the *Ae. tauschii* and *T. aestivum* BNG maps. **e**, Initial number of scaffolds in the Aet v2.0 assembly, number of scaffolds after resolution of misassembled scaffolds with the aid of the AL8/78 BNG contigs, and number of super-scaffolds after stitching. **f**, Reductions in the numbers and lengths (bp, in parentheses) of unordered super-scaffolds after alignments on only AL8/78 BNG contigs (step 1) and subsequent alignment of those from step 1 on the CIae23 BNG contigs and the Chinese Spring (CS) BNG contigs (step 2). **g**, Illustration of synergy among three BNG maps in super-scaffold stitching, anchoring, and orienting on the genetic/physical map. Two sequence super-scaffolds (s-scflds), shown at the bottom in light blue, were anchored on the genetic map of chromosome 2D by virtue of single nucleotide polymorphism (SNP) markers (AT2D1510 at 101.296 cM and AT2D1438 at 101.25 cM). S-scfld109859 included both SNP markers and thus was located, ordered, and oriented on the genetic map of chromosome 2D. However, s-scfld109830 with only one SNP marker, which co-segregated with other markers, could not be ordered or oriented on the genetic map. Using the AL8/78 BNG map alone (the contigs shown on top in purple colour), two of its contigs, ctg5441 and ctg1222, could be aligned to s-scfld109859 and contig ctg4835 could be aligned to s-scfld109830, but the two s-scflds were not connected. It is only when the second *Ae. tauschii* BNG map (CIae23) was deployed that the two super-scaffolds could be linked. CIae23 BNG ctg54 (at centre in green) bridged the gap between them and they were located, ordered, and oriented. The red lines join corresponding Nt.BspQI sites in the BNG contigs and s-scflds.

The *Ae. tauschii* AL8/78 genome sequence was assembled in five steps (Extended Data Fig. 1a). The core was assembly Aet v1.1 (Extended Data Fig. 1b) based on sequences of 42,822 bacterial artificial chromosome (BAC) clones. This assembly was merged with a whole-genome shotgun (WGS) assembly (Aet WGS 1.0) and WGS Pacific Biosciences mega-reads^6^ to extend scaffolds and close gaps, thereby producing assembly Aet v2.0 (Extended Data Fig. 1b, c). Misassembled scaffolds were detected with the aid of an AL8/78 optical BioNano genome (BNG) map and resolved, producing assembly Aet v3.0 (Extended Data Fig. 1d, e). Two additional BNG maps were constructed and, along with the genetic and physical maps^7^, used in super-scaffolding and building pseudomolecules for the final assembly, Aet v4.0 (Extended Data Fig. 1d, f, g).

**Extended Data Figure 2:**
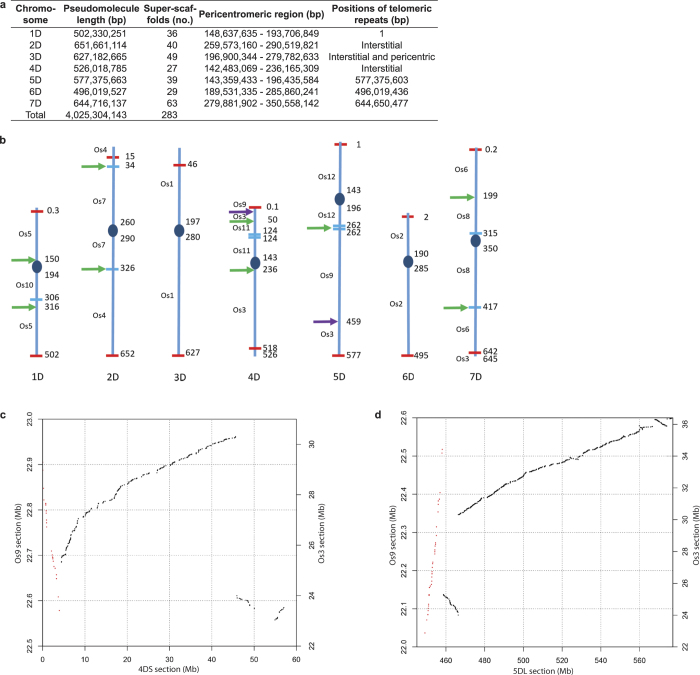
Assembly and characteristics of pseudomolecules. **a**, Characterization of pseudomolecules: their length in base pairs, the number of super-scaffolds per pseudomolecule, the locations of centromeric regions with uncertain ordering of scaffolds and super-scaffolds, and the locations of arrays of telomeric repeats. The locations in base pairs are given for the arrays of telomeric repeats that are terminally located on the pseudomolecules. **b**, The locations on the *Ae. tauschii* pseudomolecules of the termini of the ancient chromosomes involved in NCIs or end-to-end fusions (green arrows). The current locations of the ancient termini on the *Ae. tauschiii* pseudomolecules are indicated by blue horizontal bars. Also indicated by blue horizontal bars are the locations of termini of the chromosomes involved in end-to-end chromosome fusions. The current locations of termini of the ancient chromosomes that were the recipient chromosomes in NCIs are indicated by red horizontal bars. Each *Ae. tauschii* pseudomolecule starts at the tip of the short arm (top) and ends with a nucleotide, rounded to megabases, at the tip of the long arm (bottom). An ancient terminus (red bar) with a location other than one of these two numbers or not matching the position of a green arrow is at an ectopic site. Purple arrows indicate the breaks of the 4DS–5DL reciprocal translocation. The positions of pericentromeric regions with uncertain super-scaffold ordering are indicated in megabases flanking the centromeres (ovals). All measures are given in megabases (to the right of the chromosomes). Note that the locations of eight ancient termini of the recipient chromosomes (red bars) and two ancient termini of the inserted chromosomes (blue bars) are not at the tips of chromosomes or NCI sites. In addition, four ancient termini are end-to-end fused (double blue bars). Thus, 14 out of 23 sites (synteny of the short arm of chromosome Os10 inserted in Os5, making up chromosome 1D, is too poor to allow reliable determination of the terminus) are at ectopic locations. **c**, Dot plot of the distal end of *Ae. tauschii* chromosome arm 4DS (horizontal axis) and a section of rice chromosome Os9 (left vertical axis) and a section of rice chromosome Os3 (right vertical axis). The red dots are 4D genes homologous to genes on Os9 and the black dots are 4D genes homologous to genes on Os3. **d**, Dot plot of the distal end of *Ae. tauschii* chromosome arm 5DL (horizontal axis) and a section of rice chromosome Os9 (left vertical axis) and a section of rice chromosome Os3 (right vertical axis). The red dots are 5D genes homologous to genes on Os9 and the black dots are 5D genes homologous to genes on Os3. Together, **c** and **d** show that both 4D and 5D have contiguous segments of a chromosome corresponding to Os9 and both also have contiguous segments of a chromosome corresponding to Os3. Therefore, the Os9/Os3 translocation present in 4D and 5D is reciprocal.

The combined length of the pseudomolecules was 4,025,304,143 bp, and they contained 95.2% of the sequence (Extended Data Fig. 2a). About 200 Mb of the super-scaffolds remained unassigned and 76 Mb of the AL8/78 BNG contigs were devoid of aligned super-scaffolds. We conclude therefore that the size of the *Ae. tauschii* genome is about 4.3 Gb.

To assess the accuracy of our assembly, sequences of 195 independently sequenced and assembled AL8/78 BAC clones^8^, which contained 25,540,177 bp in 2,405 unordered contigs, were aligned to Aet v3.0. Five contigs failed to align and six extended partly into gaps, accounting for 0.25% of the total length of the contigs. Seven BAC contigs, although aligned, contained internal structural differences. The remaining contigs aligned end-to-end with an average identity of 99.75%. Considering the likely possibility that errors existed in both assemblies, this validation indicated that our assembly is remarkably accurate.

More work is nevertheless needed to order super-scaffolds in the pericentromeric regions of the pseudomolecules (Extended Data Fig. 2a). The ends of the pseudomolecules also need attention. Arrays of the rice (*Oryza sativa*) telomeric repeat^9^ (TTTAGGG) were detected in all pseudomolecules and in three of the unassigned scaffolds, but in only four pseudomolecules was such an array terminally located (Extended Data Fig. 2a) and presumably marked a telomere.

The wheat and *Aegilops* genomes have seven chromosomes, which evolved by dysploid reductions from twelve ancestral chromosomes^10^. The dominant form of dysploid reduction in the grass family is the nested chromosome insertion (NCI), in which a chromosome is inserted, usually by its termini, into the centromere-adjacent region of another chromosome^11^. *Ae. tauschii* chromosomes 1D, 2D, 4D, and 7D originated by NCIs (Extended Data Fig. 2b). Chromosome 5D probably originated by an end-to-end fusion of the short arms of ancestral chromosomes corresponding to rice chromosomes Os9 and Os12, followed by a reciprocal translocation involving the Os9 section of 5DL and the Os3 section of 4DS (Extended Data Fig. 2b–d).

A nearly perfect array of telomeric repeats 141-bp long was located on *Ae. tauschii* chromosome 2D at 33,306,010 to 33,306,151 bp, which is within a 2D NCI site (31,408,197 to 34,183,608 bp, Supplementary Data 1), indicating that telomeric repeats may be directly involved in the NCI process. An NCI may be a special case of telomere-driven ectopic recombination. Of the 23 ancestral chromosome termini that we could study by investigating the colinearity of *Ae. tauschii* pseudomolecules with those of rice, 14 (61%) have been involved in one or more inversions or end-to-end fusions (Extended Data Fig. 2b).

**Extended Data Figure 3:**
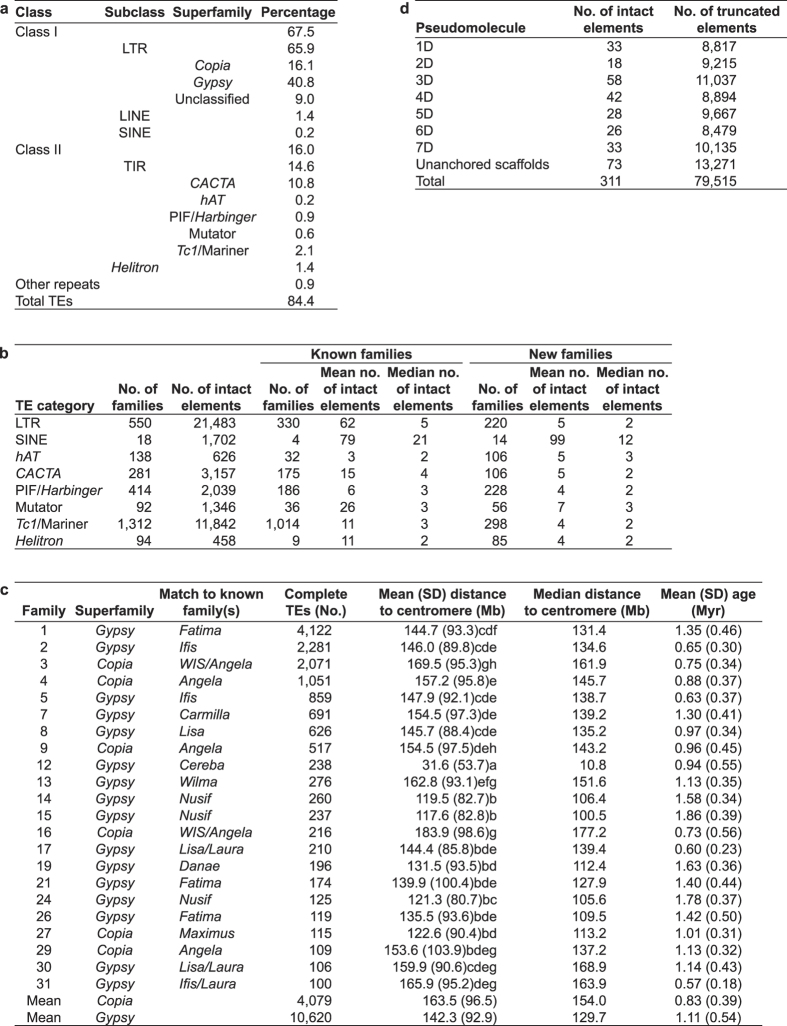
*Ae. tauschii* TEs. **a**, Percentages of the total *Ae. tauschii* DNA represented by various types of TEs. LINE, long interspersed nuclear element. **b**, Comparison of the numbers of TE families newly discovered in the *Ae. tauschii* genome with the numbers of already known TE families present in the *Ae. tauschii* genome. Also shown are the numbers of complete elements in the families. The category PIF/*Harbinger* includes Tourist MITEs and category *Tc1*/Mariner includes Stowaway MITEs. **c**, The mean and median distances of the 22 most abundant LTR-RT families from the centromere in the *Ae. tauschii* pseudomolecules. SD, standard deviation. Means followed by the same letter are not significantly different from each other at the 5% significance level (one-way analysis of variance, two-sided Tukey’s test). The boundary between the proximal and distal groups was at 136 Mb from the centromere. The average age of the complete elements in 11 proximal families was 1.11 ± 0.54 Myr (mean ± SD) whereas that in the 11 distal families was 0.86 ± 0.43 Myr (two-sided *t*-test, *P* < 0.0001, *n* = 11). **d**, Distribution of complete and truncated TEs of the *Gypsy* family 12, which is homologous to barley centromeric repeat *cereba*, among the *Ae. tauschii* pseudomolecules and unanchored scaffolds.

Transposable elements (TEs) represented 84.4% of the genome sequence. By far the most abundant (65.9% of the sequence) were the long terminal repeat retrotransposons (LTR-RTs) (Extended Data Fig. 3a). *Gypsy* and *CACTA* were the most abundant RNA and DNA transposon super-families, respectively. Most of the 1,113 new TE families discovered were low in copy number, from one to three complete elements; new short interspersed nuclear element (more commonly known as SINE) families were exceptional in this respect (Extended Data Fig. 3b).

**Figure 1:**
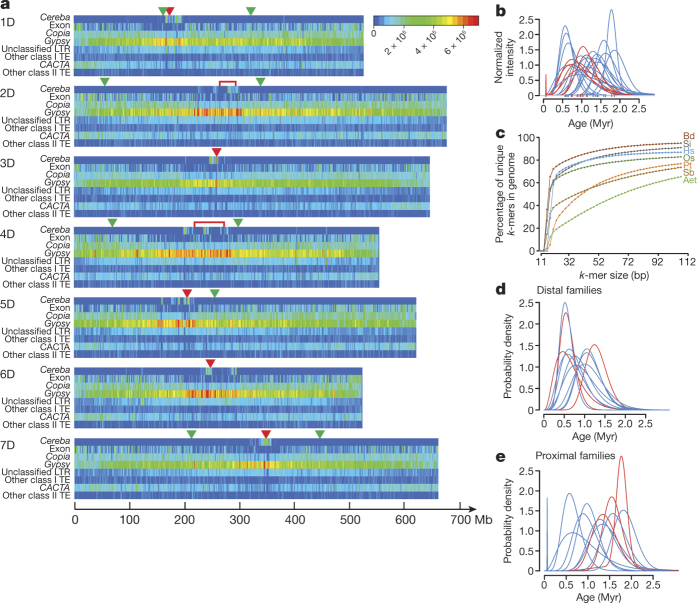
*Aegilops tauschii* TEs. **a**, Heat maps of densities along the pseudomolecules of centromeric LTR-RT *cereba*, exons, *Copia* and *Gypsy* LTR-RT super-families, unclassified LTR-RTs, other types of class I (RNA) TEs, *CACTA* (DNA) TEs, and other types of class II (DNA) transposons. Red arrowheads or brackets indicate the positions of the centromeres. Green arrowheads indicate the sites of NCIs (1D, 2D, 4D, and 7D) and chromosome (telomere) fusion (5D). **b**, Normalized insertion (intensity) rates of the 22 most abundant *Copia* (red) and *Gypsy* (blue) families in the *Ae. tauschii* genome during the past 3 Myr. **c**, The *k*-mer uniqueness ratio for *Ae. tauschii* (Aet), compared to *P. taeda* (Pt), *O. sativa* (Os), *B. distachyon* (Bd), *Setaria italica* (Si), *S. bicolor* (Sb), and *Homo sapiens* (Hs). **d**, **e**, Ages of TEs in the 11 distally located families (**d**) and the 11 proximally located families (**e**) from the 22 families in **b**. The age difference between the two groups is statistically significant (*P* = 0.003, two-sided *t*-test, *n* = 22). PowerPoint slide

The density of a pseudomolecule of the *Gypsy* super-family and unclassified LTR-RTs increased from the telomere towards the centromere whereas the density of the *Copia* and *CACTA* super-families mirrored exon density and increased in the opposite direction (Fig. 1a). Individual families often deviated from these general patterns, as indicated by the mean and median distances to the centromere of the 22 most abundant LTR-RT families (Extended Data Fig. 3c).

*Gypsy* family 12, which is homologous to the barley (*Hordeum vulgare*) centromeric retrotransposon *cereba* located at barley centromere cores^12^, clustered in the centromeric regions (Fig. 1a). We found 311 complete elements and 79,515 truncated elements (Extended Data Fig. 3d). The unassigned scaffolds contained more complete elements compared to truncated elements than the pseudomolecules, which suggests that unassigned scaffolds are enriched for centromere core sequences.

Our dating of LTR-RT insertions suggested a TE amplification peak about 1 million years ago (Ma). Accepting this result at its face value neglects amplification dynamics in individual families^13^ and TE removal by deletions^14^. Modelling the demography of LTR-RTs on the basis of the rates of TE ‘births’ and ‘deaths’ in individual families showed that LTR-RT families were subjected to sequential bursts of amplification followed by silencing over the past 3 million years (Myr) (Fig. 1b). TEs older than 3 Myr were mostly absent, which is consistent with fast turnover of intergenic DNA in Triticeae genomes^15^.

Other evidence for the very high rate of replacement of TEs in the *Ae. tauschii* genome was provided by *Ae. tauschii k*-mer uniqueness ratio analysis (Fig. 1c). The ratio is the percentage of the genome that is covered by unique sequences of length *k* or longer^16^. The *Ae. tauschii* ratio was the lowest among the seven genomes compared in Fig. 1c, including the much larger pine (*Pinus taeda*) genome. Not only is the *Ae. tauschii* genome exceptionally repetitive, but also its TEs are highly similar to each other.

LTR-RT families in the proximal chromosome regions were older on average than families in the distal chromosome regions (Fig. 1d, e). This is most likely caused by faster deletion of DNA from distal chromosome regions than from proximal chromosome regions^13,17,18^.

**Extended Data Figure 4:**
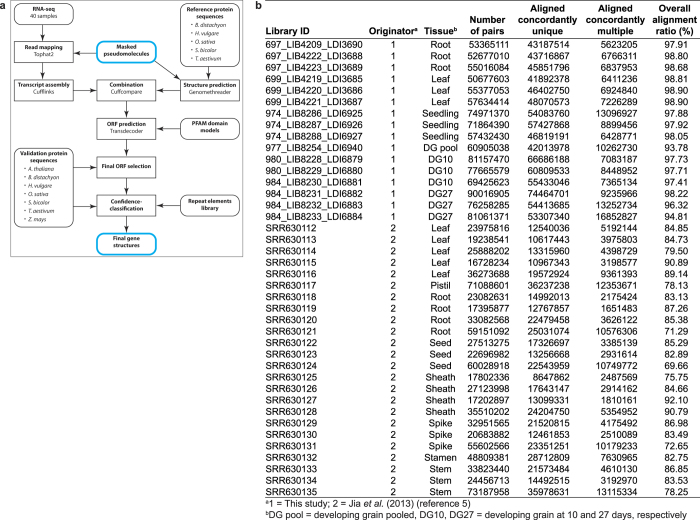
*Ae. tauschii* gene annotation. **a**, Protein-coding gene annotation workflow. Rectangles indicate tools used, rounded rectangles indicate sources of data, and the blue rounded rectangles indicate outputs. **b**, *Ae. tauschii* accession AL/8/78 RNA-seq datasets used in gene annotation.

**Extended Data Figure 5:**
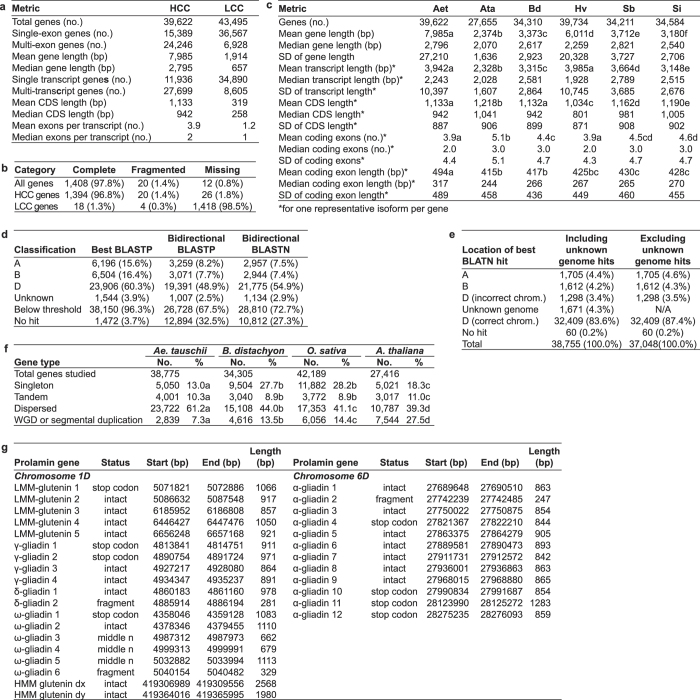
*Ae. tauschii* genes. **a**, Characteristics of HCC and LCC protein-coding genes and pseudogenes annotated in Aet v4.0. **b**, Numbers and percentages of the 1,440 BUSCO genes found among the 83,117 annotated genes. **c**, Comparison of HCC genes annotated in *Ae. tauschii* with those annotated in *A. thaliana* araport11, Ata; *B. distachyon* v3.1, Bd, *H. vulgare* HC IBSCv1.0, Hv; *S. bicolor* v2.1, Sb; and *S. italica* v2.1, Si. For rows with means, those followed by the same letter do not significantly differ at the 5% significance level (two-tailed *z*-test, Bonferroni adjusted). **d**, Numbers and percentages of best BLASTP (protein), bidirectional BLASTP, and bidirectional BLASTN (nucleotide) hits for 39,622 *Ae. tauschii* HCC genes against gene models in the A, B, and D genomes of wheat (Chinese Spring), in unassigned wheat scaffolds, and those with no hits, using a threshold *E*-value of 10^−5^. **e**, Numbers and percentages of best BLATN hits for nucleotide sequences of the 38,775 *Ae. tauschii* HCC genes located on the *Ae. tauschii* pseudomolecules against the nucleotide sequences of the wheat A-, B-, and D-genome (Chinese Spring) scaffolds. Unassigned scaffolds (unknown genome) were either included (middle) or excluded (right) from the calculations. Lower numbers of best BLAST hits with gene models as targets compared to BLAT hits with nucleotide sequences as targets (*P* < 0.0001, two-sided *χ*^2^ test, *n* = 23,906 and 38,775) are caused by annotation differences between the *Ae. tauschii* genome and the wheat D genome and by the inability to separate hits of orthologues from hits of paralogues. **f**, Classification by the MCScanX program of the *Ae. tauschii, B. distachyon*, rice, and *A. thaliana* (TAIR10) HCC genes located on pseudomolecules according to their copy number (single or duplicated) and the type of duplication. Dispersed duplicated genes included non-tandem genes duplicated both on the same chromosome and different chromosomes but did not include genes duplicated by the WGD or genes in segmental duplications. The same setting of the MCScanX program was used to detect and classify duplicated genes in the four genomes. Percentages followed by the same letter do not significantly differ at the 5% significance level *(χ*^2^-test, two-tailed, Bonferroni adjusted, *n* = total numbers of genes in each species). **g**, Prolamin genes annotated in the *Ae. tauschii* pseudomolecules 1D and 6D and their characteristics. LMM, low molecular mass; HMM, high molecular mass.

Our annotation pipeline (Extended Data Fig. 4a, b) annotated 83,117 genes in Aet v4.0 and allocated 39,622 of them into the high-confidence class (HCC) (gene set v2.0) and the remaining 43,495 into the low-confidence class (LCC) (Extended Data Fig. 5a). Of the HCC genes, 38,775 were in the pseudomolecules and 847 (2.2%) were in unassigned scaffolds. The total length of predicted HCC genes was 316,517,346 bp (7.5%) and the total length of their mRNAs was 145,062,217 bp (3.4%). Gene annotation was validated by a search for 1,440 BUSCO genes^19^, of which 1,408 (97.8%) were correctly predicted among the 83,117 genes (Extended Data Fig. 5b). *Ae. tauschii* genes were compared with genes annotated in four grass genomes and the *Arabidopsis thaliana* genome (Extended Data Fig. 5c). *Ae. tauschii* genes were the longest, had the longest mean exon length, and together with barley genes had the longest transcript lengths among the genomes. Otherwise, they were similar to genes in the other genomes, except for having a lower average number of exons.

**Figure 2:**
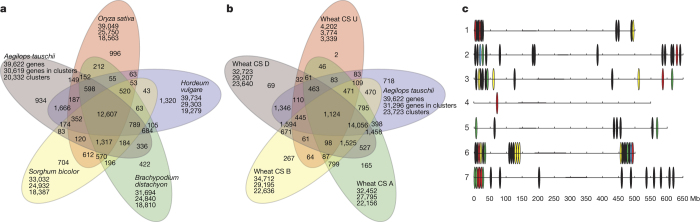
*Ae. tauschii* genes. **a**, OrthoMCL gene family clustering of the *Ae. tauschii* HCC genes with those of *S. bicolor*, *B. distachyon*, *O. sativa*, and *H. vulgare*. The first number below each species name is the total number of genes analysed, the second number is the number of genes in clusters, and the third number is the number of gene clusters. Numbers in the sections of the diagram indicate the numbers of clusters (gene groups), not individual genes. **b**, OrthoMCL clustering of *Ae. tauschii* HCC genes with hexaploid wheat cv. Chinese Spring (CS) genes classified by A-, B-, and D-genome origin or unclassified origin (U). **c**, Distribution of RGA multi-gene loci (filled ovals) along the seven *Ae. tauschii* pseudomolecules including coiled-coil (CC)–NBS–leucine-rich repeats (LRRs) (red ovals), receptor-like kinases (black ovals), receptor-like proteins (yellow ovals), NBSs (blue ovals), and NBS–LRRs (green ovals). Centromeric regions are indicated by the thicker horizontal line for each pseudomolecule. A locus was considered to be multi-gene if it contained at least three genes of a specific RGA class with a maximum distance between genes of 300 kb. PowerPoint slide

The *Ae. tauschii* HCC genes and HCC genes annotated in the barley, *Brachypodium distachyon*, rice, and sorghum (*Sorghum bicolor*) genomes were clustered into gene families and compared (Fig. 2a). The genomes contained between 422 and 1,320 genome-unique clusters and shared 12,607 clusters, probably representative of the gene core of grass genomes. The *Ae. tauschii* and barley HCC gene sets exclusively shared 1,666 clusters, likely to be representative of gene families unique to Triticeae.

A similar comparison was made with genes annotated in hexaploid wheat cv. Chinese Spring^20^ (Fig. 2b). The numbers of genome-unique clusters were lower in the wheat genomes than in the *Ae. tauschii* genome, which may reflect the phylogenetic proximity of the genomes and the correlation between the number of genome-unique clusters and the numbers of genes annotated in the genome (*r* = 0.85 and 0.98, see Methods). The *Ae. tauschii* and wheat genomes shared 15,180 clusters, probably representative of the gene core of the wheat and *Aegilops* genomes. The wheat B genome shared more clusters exclusively with the *Ae. tauschii* genome and with the wheat D genome than did the A genome.

Several BLAST or BLAT search approaches were employed to estimate the numbers of gene differences between the *Ae. tauschii* AL8/78 genome and Chinese Spring wheat D genome (Extended Data Fig. 5d, e and Supplementary Data 2). If the orthologue for an *Ae. tauschii* gene is absent from the wheat D genome, the best search hit will most likely be a paralogue in any of the three wheat genomes or an orthologue in the A or B genomes. Large percentages of such hits were observed (Extended Data Fig. 5d, e). They exceeded the 0.17 and 0.27% of genes previously estimated to have been deleted from the wheat D genome since the origin of hexaploid wheat^17,21^ by nearly two orders of magnitude. Bidirectional BLAST searches showed additional reductions in successful searches, indicating the absence of orthologues in both the wheat D genome and the *Ae. tauschii* genome and differences in gene annotation in these two genomes (Extended Data Fig. 5d). Only 87.4% of *Ae. tauschii* genes were correctly located by BLATN searches in the D genome (Extended Data Fig. 5e), further highlighting the magnitude of polymorphism for gene presence and absence (copy number variation) in these genomes.

Some of the *Ae. tauschii* genes and gene clusters classified as unique or Triticeae specific may be of practical importance. Prolamin genes, which represent several seed-storage protein families unique to Triticeae^22^, are central to the bread-making properties of wheat flour. We discovered and characterized 31 prolamin genes in the *Ae. tauschii* genome sequence (Extended Data Fig. 5g).

**Extended Data Figure 6:**
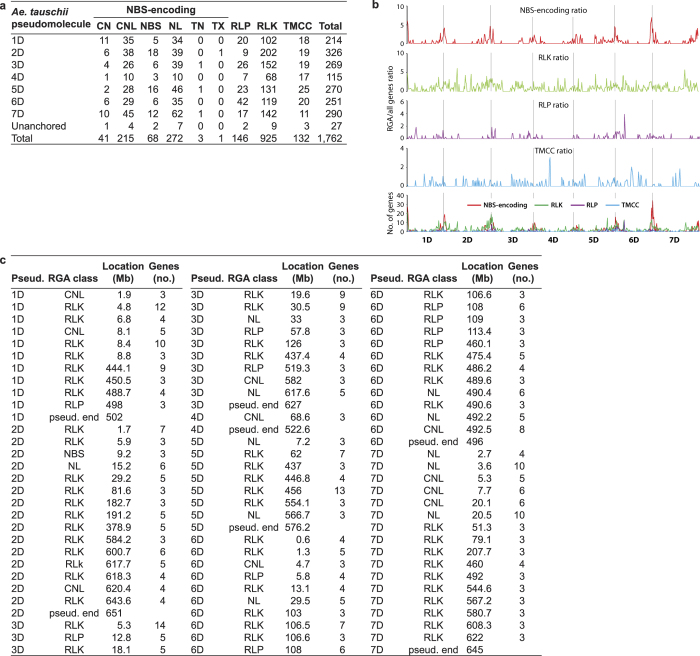
Resistance gene analogues. **a**, The numbers of predicted RGAs in the *Ae. tauschii* genome. CN, CC–NBS; CNL, CC–NBS–LRR; NL, NBS–LRR; RLK, receptor-like protein kinase; RLP, receptor-like protein; TMCC, transmembrane coiled-coil protein; TN, TIR–NBS; TX, TIR–unknown. **b**, Distribution of RGAs along the *Ae. tauschii* pseudomolecules. The bottom graph shows absolute numbers of genes homologous to nucleotide-binding site–leucine-rich repeat (NBS-encoding), receptor-like protein kinase (RLK), receptor-like protein (RLP), and transmembrane coiled-coil protein (TMCC) along each of the seven pseudomolecules oriented with the tip of the short arm to the left. The top four graphs show the ratio of the number of genes in each RGA class to the total number of genes in a sliding window of 10 Mb. This quotient removes the effects of gene density variation along the chromosome from the data and shows that NBS–LRRs, but not the remaining four classes of RGA, are disproportionally more abundant in the distal regions of the *Ae. tauschii* chromosomes than in the proximal regions. **c**, Locations of RGA multi-gene loci on the pseudomolecules. The dominant RGA gene class is indicated for each locus. CNL, CC–NBS–LRR; NL, NBS–LLR; pseud., pseudomolecule; RLK, receptor-like protein kinase; RLP, receptor-like protein.

Another class of *Ae. tauschii* genes that are of practical importance are disease resistance genes. Using a disease resistance gene analogue (RGA) prediction pipeline^23^, we annotated 1,762 RGAs in the *Ae. tauschii* genome sequence (Extended Data Fig. 6a) and list them in Supplementary Data 3. Nucleotide-binding site (NBS)-type RGAs tend to cluster near the ends of chromosomes (Extended Data Fig. 6b). That is true for RGA multi-gene loci in general. They show a distinct preference for distal chromosome regions (Fig. 2c). A total of 81 RGA multi-gene loci were identified (Extended Data Fig. 6c). The largest number was in 6D (20 loci), followed by 7D and 2D (16 and 15 loci, respectively), and the smallest number was in 4D (1 locus).

Of the 38,775 HCC genes located on the pseudomolecules, only 5,050 (13.0%) were single-copy genes. By far the most abundant were dispersed duplicated genes (Extended Data Fig. 5f). These were disproportionally more abundant in the *Ae. tauschii* genome than in the genomes of *B. distachyon*, rice, or *A. thaliana.* A remarkable example of the abundance of dispersed duplicated genes in the *Ae. tauschii* genome is provided by the *GLI1–GLU3* gene region on 1D. The region comprises 13 genes in the *B. distachyon*, rice, and sorghum genomes but it contains over 80 additional genes in the *Ae. tauschii* genome, most of them dispersed duplicated genes^22^.

**Figure 3:**
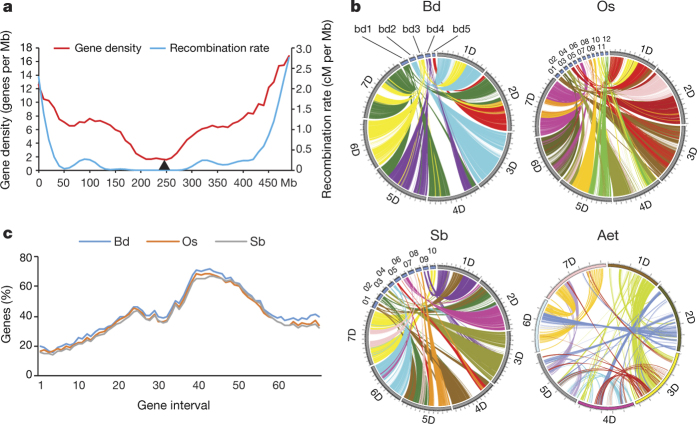
Recombination rates, gene density, and gene colinearity. **a**, Relationship between gene density (red) and meiotic recombination rate (blue) for pseudomolecule 6D. The black triangle depicts the centromere. HCC gene set v1.0 was used to generate this figure. A similar pattern is observed in figures generated with HCC gene set v2.0 (Extended Data Fig. 7a). **b**, Synteny between the *Ae. tauschii* pseudomolecules and those of *B. distachyon* (Bd), rice (Os), sorghum (Sb), and self-syntenic blocks within the *Ae. tauschii* (Aet) genome. In the three interspecific comparisons, the absence of synteny in the centromere-adjacent regions of the *Ae. tauschii* pseudomolecules is reflected by empty space in those regions. Within the Aet genome, the largest syntenic regions are 1D with 3D and 6D with 7D. **c**, The percentage of genes in 50-gene intervals along the *Ae. tauschii* pseudomolecule 6D that are colinear with genes along homeologous pseudomolecules of *B. distachyon*, rice, and sorghum. The great similarity among the profiles indicates that they all reflect decay of colinearity along chromosome 6D. Note that the colinearity profiles are broadly an inverse of the recombination rate profile. PowerPoint slide

**Extended Data Figure 7:**
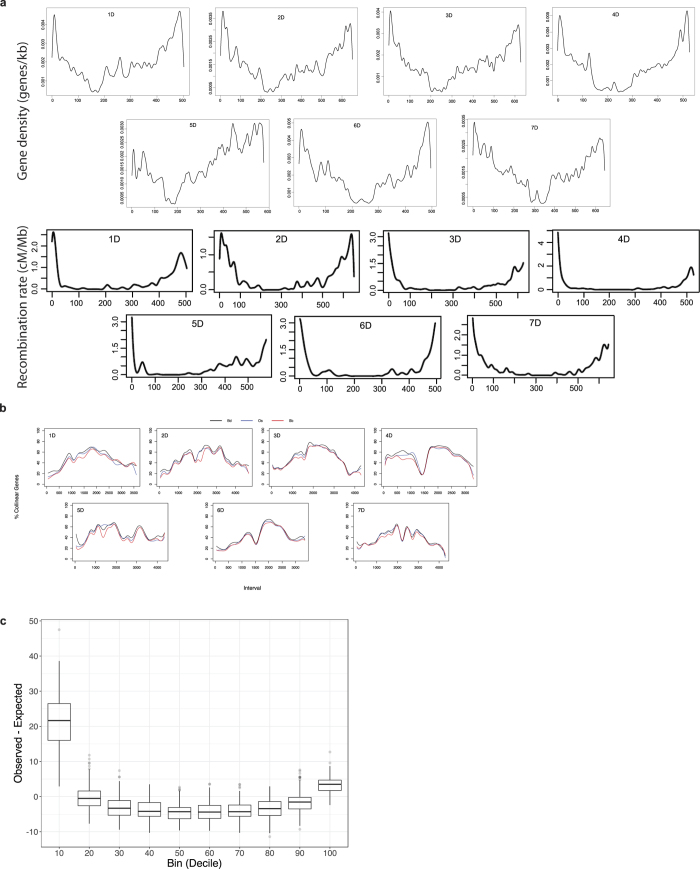
Distribution of genes, recombination rates, and synteny along pseudomolecules. **a**, Gene density (top) and recombination rates (bottom) along *Ae. tauschii* pseudomolecules. The functions are scaled so that these densities integrate to 1. Recombination rates are expressed in cM/Mb. Gene density and recombination rates were highly correlated along each pseudomolecule (*P* < 0.0001, two-tailed *t*-test,10-Mb window, *n* = 51, 63, 63, 53, 57, 50 and 65 for 1D, 2D, 3D, 4D, 5D, 6D and 7D, respectively). **b**, Quantification of gene colinearity with *B. distachyon* (Bd), rice (Os), and sorghum (Sb) pseudomolecules along *Ae. tauschii* pseudomolecules expressed as percentage of colinear genes in an interval of 50 genes (smoothed with local linear fits using bandwidth 5). Colinearity is expressed per gene not per megabase, and the profile is therefore unaffected by gene density variation along the *Ae. tauschii* chromosomes. The more conservative HCC gene set v1.0 was used to generate these colinearity graphs. The gene set v1.0 was also used to generate graphs in Extended Data Fig. 10c, and the two sets of figures are therefore comparable. **c**, Clustering of *Ae. tauschii* genes along chromosomes. In each box plot, the centre line represents the median, the lower and upper hinges represent the first and third quartiles, respectively, the whisker length is 1.5× the interquartile range, and the dots indicate extreme observations. To account for distributional changes in gene density along chromosomes, each chromosome was split into 50 segments so that each segment had roughly the same number of genes. *χ*^2^ goodness-of-fit tests for ten bins were performed for the null hypothesis that each sample of intergenic distances follows an exponential distribution, which would hold if genes were homogenously distributed along the chromosomes. The rate parameter for each segment was estimated using maximum likelihood and the bins were chosen from the deciles of the reference distribution so that each bin had the same expected count. In 340 out of 350 (97%) segments, the null hypothesis of homogeneous gene distribution was rejected. The largest factor leading to a rejection was the discrepancy between the observed and expected counts for very short distances, as visualized in the boxplots. The box plots display differences between the expected and empirical frequency of intergenic distances, in which the shortest distances and longest distances are most overrepresented in the empirical data. The shortest distances are to the left and the longest distances are to the right. The overrepresentation of short and long distances is expected if genes are clustering into insulae. Distances within gene insulae will be shorter and distances between insulae will be longer than expected on the basis of the exponential distribution, which would apply if genes were distributed homogeneously.

The density of HCC genes was highest (about 10–16 genes per Mb) in the distal chromosome regions, declining to about 2 genes per Mb in the proximal regions (Fig. 3a), and was correlated with recombination rates (Extended Data Fig. 7a). We provide here empirical evidence for the hypothesis that recombination rate is the causal variable in this relationship^24^. First, NCIs and end-to-end chromosome fusions reposition distal and proximal chromosome regions into new sites. This should perturb gene distribution around the NCI site^25^ but no such perturbations are apparent in the *Ae. tauschii* pseudomolecules (Fig. 1a). We propose that after an NCI or end-to-end fusion the repositioned region acquires a new recombination rate conforming to its new position relative to the telomere^26^. The new recombination rate alters the rate with which dispensable DNA is deleted from the region^17^, thereby altering gene density. Second, chromosome 6D is one of two *Ae. tauschii* chromosomes that have not been involved in an NCI or end-to-end chromosome fusion (Extended Data Fig. 2b). The gene density and recombination rate profiles show collocated major and minor peaks (Fig. 3a). Our proposal of recombination rate as the causal variable readily accounts for the peaks in both profiles. The major peaks in the recombination rate profile are caused by the preference of first crossovers for distal chromosome regions and the minor peaks are caused by the displacement of the rare second crossovers by crossover interference into the proximal regions. The peaks in gene density will evolve by the first process described above. However, if we assume that gene density is the causal variable, we can find no explanation for the evolution of the peaks.

The analysis of the distribution of HCC genes along the pseudomolecules allows us to extend our hypothesis that *Ae. tauschii* genes occur in small clusters (insulae)^27^, which was previously based on the sequencing of several regions of the *Ae. tauschii* genome, to encompass the entire genome. We rejected the null hypothesis that genes are uniformly distributed along the chromosomes with *P* < 0.001 (Extended Data Fig. 7c), and confirmed that the increase in gene density in distal regions is primarily caused by shortening of the inter-insular distances^27^.

**Extended Data Figure 8:**
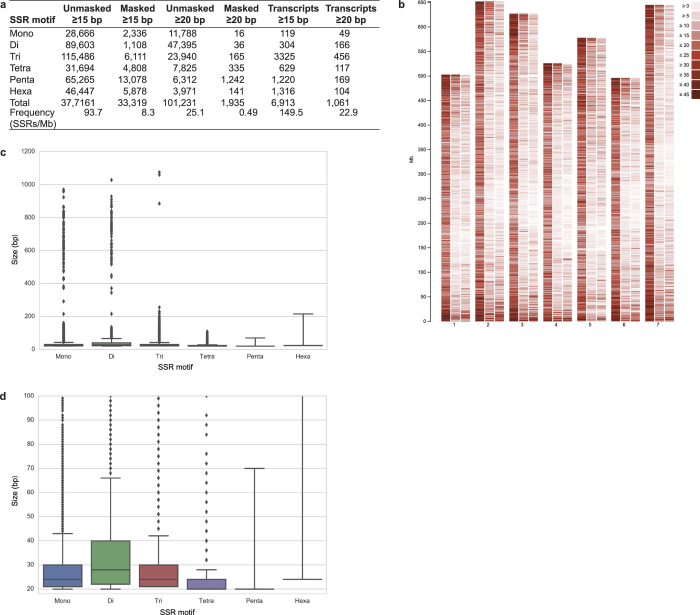
SSR frequencies and the distribution of SSR motifs in the *Ae. tauschii* genome. **a**., Summary of SSR frequencies and distribution of SSR motifs in the *Ae. tauschii* unmasked and repeat-masked pseudomolecules and transcripts. **b**, Heat maps of the density of SSRs and genes along the unmasked and repeat-masked pseudomolecules. The first column in each pseudomolecule triplet displays the density of SSRs ≥ 20 bp discovered in unmasked pseudomolecules, the second displays the density of SSRs ≥ 15 bp discovered in repeat-masked pseudomolecules, and the third displays gene density. Each column is oriented with the short arm of the pseudomolecule at the bottom. In the unmasked genome sequence, SSRs were more abundant in the distal, high-recombination regions than in the proximal, low-recombination regions and their density was correlated with recombination rate (for SSRs ≥ 20 bp: *r* = 0.86, *n* = 51; *r* = 0.73, *n* = 66; *r* = = 0.91, *n* = 63; *r* = 0.82, *n* = 53; *r* = 0.82, *n* = 58; *r* = 0.78, *n* = 50; and *r* = 0.85, *n* = 65; for pseudomolecules 1 to 7, respectively; *P* = 0.0001, two-tailed *t*-test). **c**, **d**, Box plots at two scales illustrating the frequency of sizes of the SSR motifs (≥20 bp; mono-, di-, tri-, tetra-, penta-, and hexanucleotide motifs) in the unmasked pseudomolecules. The full range of sizes from 0 to 1,200 bp is shown in **c**, whereas the 20–100-bp subset is shown in **d** to help reveal greater detail about the median and quartiles for each motif type. In each box plot, the central line represents the median, the lower and upper hinges are the first and third quartiles, respectively, and the whisker lengths indicate minimum and maximum values, excluding the extreme observations (values 1.5 times greater than the value of the third quartile).

A large number of simple sequence repeats (SSRs) were discovered in the *Ae. tauschii* genome (Extended Data Fig. 8a, c, d), and represent a resource for the development of SSR markers for wheat genetics and breeding. We designed a portal (http://aegilops.wheat.ucdavis.edu/ATGSP/data.php, at the ‘SSR search database’ link) to allow users to search for SSRs in the *Ae. tauschii* genome sequence, facilitating the development of SSR markers. In the unmasked genome sequence, SSRs were more abundant in the distal, high-recombination regions than in the proximal low-recombination regions and their density correlated with recombination rates (Extended Data Fig. 8b).

**Extended Data Figure 9:**
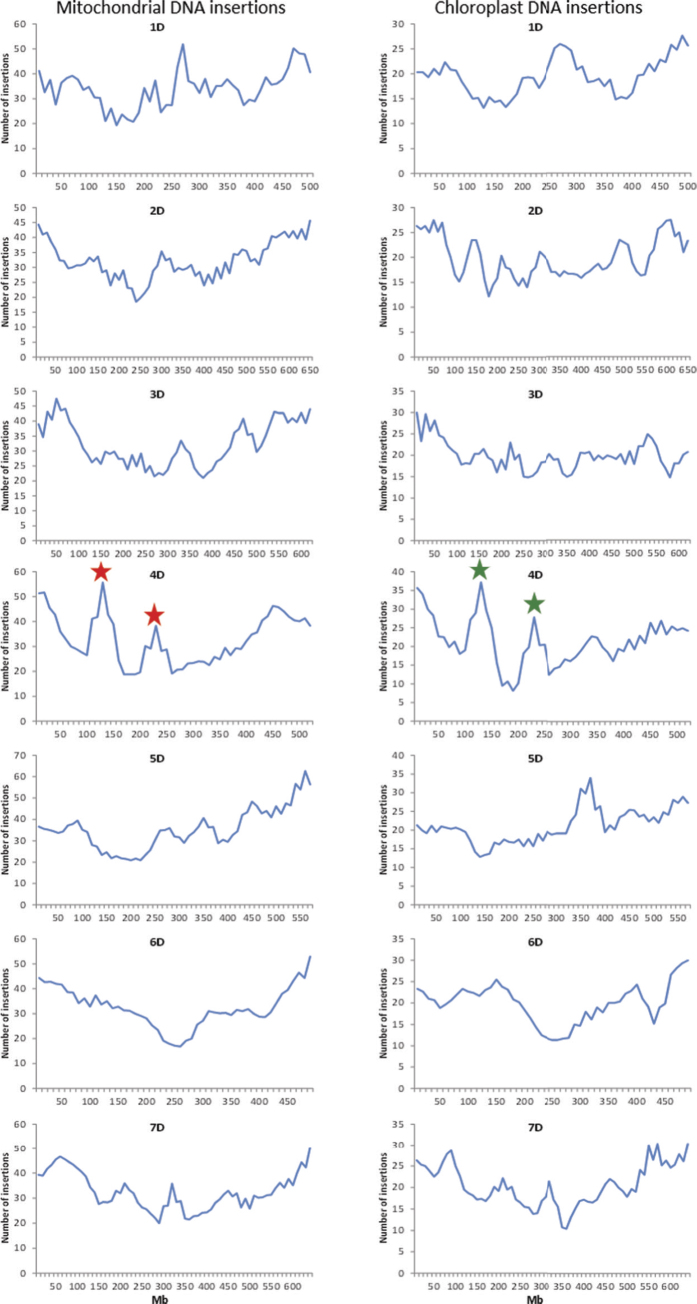
Organellar DNA insertions into the nuclear genome of *Ae. tauschii*. Horizontal axes represent the *Ae. tauschii* pseudomolecule coordinates (Mb). Sharp peaks, as exemplified by the two mitochondrial peaks for 4D marked with a red star, are likely caused by an insertion of single large segment of mitochondrial DNA that was later highly reorganized rather than by numerous independent insertions. Where there is homology between the mitochondrial and chloroplast genomes, insertion peaks are found at the same locations in the mitochondrial and chloroplast DNA insertion profiles (for example, those chloroplast peaks marked with a green star for 4D). To correlate chloroplast DNA and mitochondrial DNA insertions with recombination rates, Pearson’s correlation coefficients were calculated using the mean recombination rate in each 10-Mb interval and the number of insertions in each 10-Mb interval as variables (sample sizes are indicated in the legend of Extended Data Fig. 7a). Correlation coefficients (*r*) for individual *Ae. tauschii* chromosomes ranged from 0.41 to 0.724 (*P* = 0.002 to 0.0001, two-tailed *t*-tests) for chloroplast DNA and from 0.48 to 0.80 (*P* = 0.0002 to 0.0001, two-tailed *t*-tests) for mitochondrial DNA.

About 0.06% and 0.19% of the *Ae. tauschii* genome sequence consisted of chloroplast DNA and mitochondrial DNA insertions, respectively. These insertions correlated with recombination rates (Extended Data Fig. 9).

The *Ae. tauschii* pseudomolecules showed synteny with those of *B. distachyon*, rice, and sorghum, except for the pericentromeric regions (Fig. 3b), possibly reflecting rapid rearrangements of heterochromatic centromeric DNA in the *Ae. tauschii* genome, as previously reported for the sorghum genome^28^. Short self-syntenic blocks were detected within the *Ae. tauschii* genome, undoubtedly reflecting the pan-grass whole-genome duplication (WGD)^29^ (Fig. 3b) and comprised 2,839 (7.3%) HCC genes (Extended Data Fig. 5d).

**Extended Data Figure 10:**
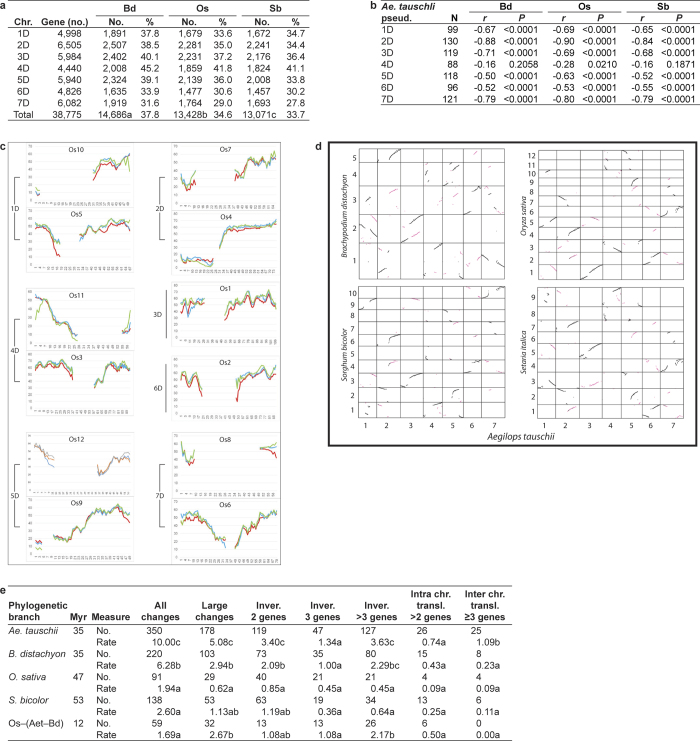
Synteny analysis. **a**, Numbers and percentages of *Ae. tauschii* genes colinear with genes along the *B. distachyon* (Bd), rice (Os), and sorghum (Sb) pseudomolecules. Numbers in the bottom row sharing the same letter do not significantly differ at the 5% significance level (two-tailed *t*-test with Bonferroni correction, *n* = 7). **b**, Number of 50-gene intervals (*N*) and correlation coefficients (*r*) and their *P* values between the numbers of colinear genes in the *Ae. tauschii* pseudomolecules with those in the *B. distachyon*, rice, and sorghum pseudomolecules and recombination rates along the *Ae. tauschii* chromosomes (two-tailed *t*-tests). The correlations between the variables are highly significant for all chromosomes except for chromosome 4D. **c**, Profiles of gene colinearity along rice pseudomolecules and the *Ae. tauschii* (red lines), *B. distachyon* (blue lines), and sorghum (green lines) pseudomolecules. The *Ae. tauschii* high-confidence gene set v1.0 was used to generate these graphs. Genes along the 12 rice pseudomolecules were used as queries in BLAST searches. The units along the horizontal axes are 50-gene non-overlapping intervals along pseudomolecules and the units along the vertical axis are the percentages of colinear genes. Because the intervals have constant numbers of genes, the data are unaffected by potential variation in gene density along rice chromosomes. The pericentromeric regions, which showed poor synteny, were not used and are presented as gaps in the profiles. The rice chromosomes that correspond to the ancestral chromosomes that were involved in NCIs in the *Ae. tauschii* genome are joined by brackets. Note that the *Ae. tauschii* profile is usually the lowest one, indicating that *Ae. tauschii* has the lowest colinearity with the rice pseudomolecule among the target genomes. This holds true for large portions of the *Ae. tauschii* chromosomes. **d**, Synteny dot plots between *Ae. tauschii* pseudomolecules (horizontal axes) and pseudomolecules of *B. distachyon*, *O. sativa*, *S. bicolor*, and *S. italica* (vertical axes). The short arms are to the left for each *Ae. tauschii* pseudomolecule. Note the similarity of structural changes shared by the four synteny comparisons. They indicate that most of the observed changes have taken place in the *Ae. tauschii* lineage. **e**, Numbers and rates per million years of inversions and interstitial and inter-chromosomal translocations involving the indicated number of genes. Large structural changes are defined as inversions (inver.) of >3 genes, intra-chromosomal translocations (intra chr. trans.) of >2 genes, and inter-chromosomal translocations (inter chr. transl.) of ≥3 genes. Os–(Aet–Bd) signifies the Pooideae branch preceding the Triticeae–Brachypodieae split. Rates followed by the same letter do not differ at the 5% significance level (paired two-tailed *t*-test, Bonferroni adjusted, *n* = 7).

Colinearity of the *Ae. tauschii* HCC genes with genes along the *B. distachyon*, rice, and sorghum pseudomolecules ranged from 37.8% with *B. distachyon* to 33.7% with sorghum (Extended Data Fig. 10a). Colinearity was the highest (about 60–70% of the HCC genes) in proximal, low-recombination regions of the *Ae. tauschii* pseudomolecules, and the lowest (about 10–20% of the HCC genes) in distal, high-recombination regions (Fig. 3c and Extended Data Fig. 7b); colinearity and recombination rate were negatively correlated in all chromosomes except for 4D (Extended Data Figs 7a, b and 10b).

To study further the influence of recombination on gene colinearity, colinearity profiles of rice pseudomolecules with those of *Ae. tauschii*, *B. distachyon*, and sorghum were developed. We predicted that the profiles would differ from the profiles obtained for *Ae. tauschii* because recombination rates along the rice chromosomes^30^ do not show the U-shape distribution characteristic of the *Ae. tauschii* chromosomes (Extended Data Fig. 7a). The rice colinearity profiles indeed differed. They showed minimal colinearity in proximal regions and colinearity either increased or remained constant towards chromosome termini (Extended Data Fig. 10c).

Since the divergence of Triticeae and Brachypodieae about 35 Ma^25^, the *Ae. tauschii* genome acquired 350 chromosome rearrangements, of which 178 were large (Extended Data Fig. 10e and Supplementary Data 1). Compared to the rice and sorghum genomes, the *Ae. tauschii* genome has been structurally evolving many fold faster (Extended Data Fig. 10e). The differences in the accumulation of structural changes among the genomes are also apparent in dot plots (Extended Data Fig. 10d). The *B. distachyon* lineage and the Pooideae branch preceding the Triticeae–Brachypodieae split were also relatively fast evolving, but the rate has further accelerated in the *Ae. tauschii* lineage after the Triticeae–Brachypodieae split (Extended Data Fig. 10e).

Fast structural evolution is accompanied by exceptional amounts of dispersed duplicated genes in the *Ae. tauschii* genome. It is tempting to attribute these characteristics to the large size of the *Ae. tauschii* genome and the abundance of repeated sequences^11^. In that case, conifer genomes, which are much larger than the *Ae. tauschii* genome and contain large amounts of TEs, should be even more dynamic than the *Ae. tauschii* genome. Yet, conifer genomes are known for their stability^31^. We propose that it is not the size and TE content that sets the *Ae. tauschii* genome apart from other genomes, including the large pine genome, but the exceptionally high amount of very similar TEs (Fig. 1c), which lead to frequent recombination errors and cause gene duplications and other structural chromosome changes. A result of this is fast genome evolution, particularly at the ends of the chromosomes, with an abundance of duplicated genes and fast decay of synteny.

## Methods

### Plants

Detailed information about *Ae. tauschii* accessions used in this project, their photos, taxonomy of *Ae. tauschii*, and its relationship to wheat can be found on our website (http://aegilops.wheat.ucdavis.edu/ATGSP/). In brief, *Ae. tauschii* accession AL8/78 was provided by V. Jaaska, who collected it near the Hrazdan River, Jerevan, Armenia. The accession is classified as *Ae. tauschii* ssp. *strangulata.* We selected this accession for the construction of BAC libraries and the *Ae. tauschii* physical map^7,32,33^ because of its genetic proximity to the wheat D genome^34^. The accession was also used for the construction of a genome-wide optical BioNano genome (BNG) map. We maintain this accession, which can be requested from the corresponding author (J.D.).

A more recent study of genetic relationships between *Ae. tauschii* and wheat uncovered a group of accessions in Caspian Iran that appeared even more closely related to the wheat D genome than AL8/78^35^. They belong to *Ae. tauschii* ssp. *tauschii* var. *meyeri.* We selected from this population accession CIae23^35^ for the construction of the second *Ae. tauschii* BNG map. CIae23 was made available by the US National Plant Germplasm System.

The third accession relevant to this project is wheat (*T. aestivum*) cv. Chinese Spring. We used accession DV418, which is derived from a colchicine-doubled haploid maintained by J.D. at UC Davis, and used it for the construction of a genome-wide BNG map of wheat.

### Illumina MiSeq BAC sequencing

The minimal tiling path across 3,578 BAC contigs consisted of 42,822 *Ae. tauschii* AL8/78 BAC clones^7^. The minimal tiling path clones were re-arrayed according to the chromosome and BAC contig to which they belonged. Both ends of each BAC clone were sequenced with the ABI 3730XL platform^36^. Minimal tiling path clones and 2,000 singleton BAC clones were allocated into 5,646 pools averaging eight, usually overlapping, BAC clones.

To isolate BAC DNA for sequencing, 1 ml of LB liquid medium containing chloramphenicol (12.5 μg ml^−1^) was inoculated with 10 μl of culture from a single well of the re-arrayed BAC library, and cells were grown at 37 °C for approximately 6 h while shaking. The culture was visually checked for sufficient growth and then combined with an additional 9 ml of LB liquid medium containing chloramphenicol (12.5 μg ml^−1^). The 10-ml culture was grown for another 16 h at 37 °C while shaking. The 10-ml cultures were visually checked for uniform growth by comparing them with each other.

A BAC pool was created, using cultures with sufficient turbidity, by combining six to ten, but typically eight, 10-ml cultures of overlapping clones from a single contig. The cultures that did not have sufficient turbidity were regrown. Those that repeatedly failed were replaced by alternative BAC clones while maintaining a minimal tiling path across the BAC contig. The pooled cultures were centrifuged for 15 min at 2,000*g* in a Sorvall centrifuge with a SA600 rotor and pellets were stored at −20 °C until a sufficient quantity of pools was available for DNA isolation. DNA was isolated from the BAC pools with the Qiagen midiprep kit protocol (Qiagen 12145), modified by including an ATP-dependent exonuclease (Qiagen) in the DNA digestion step before final column purification and by not using the pre-column provided in the kit. BAC pool DNA was sheared using the Covaris system (duty, 5%; intensity, 3; cycles per burst, 200; 20 s) and purified with the Qiagen MinElute PCR purification kit (Qiagen 28006).

Illumina libraries were constructed from 1 μg of sheared fragments of BAC-pool DNA with the KAPA library preparation kit (Kapa Biosystems) following the manufacturer’s protocol. The libraries were normalized and 48 BAC pools were combined for one 600-cycle sequencing run with MiSeq reagent kit v3. Each read was allocated to a pool on the basis of its index.

### Scaffold assembly

A total of 90,050 BAC-end sequences were generated, of which 80,906 contained both forward and reverse reads with lengths of at least 63 bp. These pairs were aligned to the scaffolds and used as an assembly validation step.

Each BAC pool contained Illumina MiSeq data from approximately eight BAC clones (ranging from a minimum of six to a maximum of ten). Most BAC clones overlapped (see above). The average overlap between adjacent BAC clones was ~25 kb, and the overall genomic span for each pool averaged ~1 Mb.

Prior to assembly, all reads were trimmed to remove any known Illumina vector and adaptor sequences. Bacterial plasmids, *Escherichia coli* sequences, and phage ΦX174 sequences were also removed. The pool reads were then assembled using SOAPdenovo2^37^ generating an initial set of contigs and scaffolds.

From the initial assemblies, any scaffolds with unusually low coverage were identified and removed as likely artefacts. The remaining scaffolds were aligned to each other, and those that were completely contained within other scaffolds in the same pool were removed.

To improve the pool assemblies further, several paired-end libraries from a previously published WGS dataset^5^ were used. This dataset included 84 libraries, from which we used six libraries of mate-paired reads from fragments ranging in length from 1.6 to 8.6 kb, with an average read length of 89 bp. These WGS libraries provided long-range linking information for the construction of scaffolds. The WGS reads were mapped to the initial assemblies using nucmer^38^. Mate-paired reads that mapped to a pool were added to the dataset of the pool. These augmented data were then re-scaffolded.

The total length of the scaffolds in this assembly (Aet v1.0, Extended Data Fig. 1b) was 5.79 Gb, exceeding the estimated genome size of 4.02 Gb^39^ by 44%. Assembly Aet v1.0 contained 250,177 scaffolds with an N50 scaffold size of 207,812 bp (Extended Data Fig. 1b). This included 96,546 scaffolds with a size of 2,000 bp or longer for a total length of 5.71 Gb. Pools that overlapped unambiguously with one another were merged. To merge the pools, all scaffolds of pools within a chromosome were aligned to one another using nucmer^38^ and then the minimus2 assembler, a modified version of minimus^40^ designed for merging assemblies. If BAC-end sequences (which were also mapped to chromosomes) indicated that two scaffolds should overlap, they were merged regardless of their chromosome origin. Scaffolds were considered for merging only if they overlapped by at least 2,000 bp with at least 99% identity. We chose these relatively permissive conditions for merging because we had the ability to detect misassembled scaffolds downstream by scaffold alignment on the BNG map contigs. Scaffold merging increased the N50 to 410,889 bp and decreased the total length of the assembly to 4.46 Gb. This assembly was named Aet v1.1 (Extended Data Fig. 1b).

### WGS reads and scaffolds

An independent WGS assembly was executed to increase the lengths of scaffolds and close gaps in the Aet v1.1 assembly. Five WGS genomic libraries were constructed (Extended Data Fig. 1c) and sequenced with an Illumina HiSeq 2500 sequencer at the Roy J. Carver Biotechnology Center, Urbana, Illinois, which produced 1.05 Tb of sequence. A total of 191× coverage in reads was employed in an assembly with the software package DenovoMAGIC2; no other sequence data were used in this process. The main steps of DenovoMAGIC2 (NRGene) were as follows.

(1) Read pre-processing and error correction. PCR duplicates, Illumina adaptors, and linkers were removed. Also removed were paired-end reads (PERs) that likely contained sequencing errors. These included all reads with sub-sequences of ≥23 bp not found in at least one other independent read. PERs with ≥10 bp sequence overlap were merged using FLASH41 to create ‘stitched reads’.

(2) Contig assembly. The stitched reads were used to build a de Bruijn graph^42^, from which contigs were constructed using a *k*-mer of 191 bp. By walking through the graph, the software identified non-repetitive contigs and used stitched reads to resolve repeats and extend non-repetitive sequences of contigs where possible.

These steps were similar to those in DenovoMAGIC1, which was used to assemble the PH207 *Zea mays* inbred line^43^, but with the following key differences. DenovoMAGIC2 takes advantage of improvements in Illumina sequencing technology and requires more sequencing; notably a minimum of 60× coverage in 2 × 250 bp reads versus ~20× in DenovoMAGIC1, and ~30× coverage in the longer PERs (compared to their absence in DenovoMAGIC1). Another change was the elimination of the requirement for TruSeq (or ‘Moleculo’) libraries from the data input. Importantly, the effective read length was increased by merging (when possible, based on ≥10 bp overlap) the 2 × 250bp reads from the short insert size PERs library. This merge generated relatively long, high quality reads (over 400 bp) with a coverage of about 50× genome equivalents. Another key change in the algorithm was the capability to support large *k*-mers (up to 191 bp, as used here) for the DeBrujn graph. The large *k*-mer size substantially reduced the complexity of the DeBruijn graph.

(3) Scaffold assembly. Scaffolding by DenovoMAGIC2 used the same algorithm as was used in the maize assembly^43^.

(4) Gap filling. A final step filled gaps using PER and mate-paired links, along with de Bruijn graph analysis to detect instances where a unique path of reads spanned a gap.

This WGS assembly, designated Aet WGS v1.0, contained scaffolds with an N50 of 1,098,654 bp (Extended Data Fig. 1b). NRGene performed another assembly (Aet WGS v1.1) later using another 8–10 kb mated-paired Illumina library. This assembly generated longer scaffolds (N50 = 11,362,824 bp) and these were used in super-scaffolding (see section Super-scaffolding and pseudomolecule construction).

We also used 35× *Ae. tauschii* genome coverage of long Pacific Biosciences (PacBio) WGS reads produced with the PacBio RSII platform at John Hopkins Genome Center. PacBio reads have a high error rate. Errors in the reads were corrected with 32.4× Illumina WGS reads (Extended Data Fig. 1c). These WGS hybrid mega-reads^6^ were used in scaffold merging (see below).

### Scaffold merging

The WGS assembly Aet WGS v1.0 had longer scaffolds but much shorter contigs than the BAC pool assembly Aet v1.1. We merged the Aet v1.1 and Aet WGS v1.0 assemblies in several steps as follows.

(1) The MaSuRCA genome assembler^44^ gap-closing module was used to fill intra-scaffold gaps in the Aet WGS v1.0 assembly. The gaps were filled using the same Illumina reads used to build the Aet WGS v1.0 assembly (Extended Data Fig. 1b). The MaSuRCA gap-closing module used original, untrimmed and uncorrected Illumina WGS reads to fill gaps in scaffolds. The gap-closing module^44^ maps the reads to the sequences flanking the gaps and attempts to build a unique path of *k*-mers closing each gap, varying the *k*-mer size from 21 to 127. This closed more than 300,000 gaps and reduced the number of contigs to 525,538, increasing the contig N50 size from 16.4 to 29.2 kb.

(2) Four ‘artificial’ mate-paired libraries of lengths 5, 12, 25, and 50 Kb were generated from the BAC pool assembly, Aet v1.1. Paired-end reads were produced every 500 bp across the assembly, producing 7–10 million pairs per library. These pairs were then used along with the SOAPdenovo2 scaffolder^37^ to re-scaffold the assembly from the previous step.

(3) These improved scaffolds were aligned to all scaffolds in the BAC-based assembly Aet v1.1, which had larger contigs and hence much better contiguity. These alignments identified many contigs in Aet v1.1 that spanned gaps in the WGS assembly, and we used the Aet v1.1 sequence to fill these gaps.

Steps 1–3 closed 560,000 gaps (83% of the total), reducing their number from 671,689 to 111,690 and estimated length from 178 to 49 Mb. These gap-closing steps increased the contig N50 size, from 16.4 to 88.3 kb.

(4) The dataset of hybrid PacBio/Illumina WGS mega-reads (previous section) was used in gap closing with the MaSuRCA gap-closing module. This step closed an additional 6,761 gaps, which increased the N50 contig size from 88.3 kb to 92.5 kb.

The assembly at this point contained a substantial number of small (<10 kb) scaffolds that were completely contained in other contigs or scaffolds. These were identified by aligning all scaffolds back to the assembly using bwa-mem^45^. A total of 51,520 scaffolds that were at least 99.5% identical to and contained within other scaffolds were removed. The result of this step was assembly Aet v2.0, with a scaffold N50 of 2,884,388 bp and contig N50 of 93,210 bp (Extended Data Fig. 1b).

### Optical BNG map construction

Etiolated leaves were collected from greenhouse-grown young plants of *Ae. tauschii* accessions AL8/78 and CIae23, and hexaploid wheat Chinese Spring, accession DV418, and mailed to Amplicon Express (Pullman) for isolation of high-molecular-mass DNA. The size of fragments produced by Amplicon Express ranged from 0.7 to 1 Mb, which greatly exceeded the sizes of fragments we were able to produce (0.3–0.4 Mb). These large fragments were central for the construction of BNG maps with large contig sizes and genome coverage. The nicking site frequencies for Nt.BspQI, Nb.BsmI, Nb.BbvCI, and Nb.BsrDI in the genome of *Ae. tauschii* acc. AL8/78 were estimated with the software Knickers (http://www.bnxinstall.com/knickers/Knickers.htm). Nt.BspQI (New England BioLabs) generated approximately 15 nicks per 100 kb, which was close to the optimum frequency, and was selected for further work. The DNA molecules were nicked with Nt.BspQI and the nicks were labelled according to the instructions provided with the IrysPrep Reagent Kit (BioNano Genomics), and as described previously^46^. The labelled DNA sample was loaded onto the IrysChip nanochannel array (BioNano Genomics). The stretched DNA molecules were imaged with the Irys imaging system (BioNano Genomics). Raw image data were converted into bnx files and from these, the AutoDetect software (BioNano Genomics) generated basic labelling and DNA length information. The DNA molecules in bnx format were then aligned against each other. Clusters were formed and assembled into contigs with a BioNano Genomics assembly pipeline^46,47^. The *P* value thresholds were optimized for pairwise assembly, extension/refinement, and final refinement for each genome. For each assembly, an initial assembly was produced and checked for chimaeric contigs. Assembly parameters were adjusted if necessary and assembly was repeated. Ultimately, BNG maps for *Ae. tauschii* accessions AL8/78 and CIae23, and for Chinese Spring wheat were generated.

### Validation of scaffolds with the AL8/78 BNG map and resolution of chimaeras

To compare sequence assemblies with the AL8/78 BNG map, the Aet v2.0 scaffolds were digested *in silico* with Nt.BspQI nickase using the program Knickers and aligned on the BNG map with RefAligner. The alignments were visualized in IrysView. Knickers, RefAligner, and IrysView were obtained from BioNano Genomics (https://bionanogenomics.com/support/software-downloads/). Of 111,834 scaffolds in the Aet v2.0 assembly (Extended Data Fig. 1b), 2,295 were of sufficient length to be validated by the BNG map. Of these, 120 were chimaeric and were disjoined, which increased the total number of scaffolds to 111,973 (Extended Data Fig. 1e).

### Super-scaffolding

Super-scaffolds were generated with the Stitch algorithm^48^ using the AL8/78 BNG map contigs as guides. The filtering parameters of Stitch were trained to be suitable for the *Ae. tauschii* AL8/78 genome, and Stitch was performed in iterations until no additional super-scaffolds could be produced. After each round of Stitch, the ‘potentially chimaeric scaffolds’ flagged by the program were manually checked and resolved if necessary. Stitching reduced the 111,973 scaffolds to 110,527 super-scaffolds. In the next super-scaffolding step, we used 305 scaffolds of NRGene assembly WGS v1.1, which reduced the number of super-scaffolds to 109,861 (Extended Data Fig. 1e). The total length of these super-scaffolds was 4,224,918,192 bp. This was the Aet v3.0 assembly (Extended Data Fig. 1b).

### Assessment of the accuracy and completeness of assembly Aet v3.0

Sequences of 195 BAC clones were downloaded from NCBI, where they were deposited as separate accessions^8^. The clones were originally sequenced using Sanger sequencing technology and assembled with the Celera Assembler^49^. These BAC sequences contained 2,405 contigs of a total length of 25,540,177 bp. The contigs were aligned to Aet v3.0 as follows. The contigs in the Aet v3.0 were indexed and the 2,405 contigs were aligned to the Aet v3.0 assembly using bwa in its long-read mode^45^. This procedure consistently aligned 2,180 contigs end-to-end to the contigs of Aet v3.0. The contigs were then re-aligned using nucmer^38^ with more sensitive settings, and additional end-to-end and partial alignments were identified. All alignments not matching end to end were inspected to determine whether they contained misassemblies.

### Pseudomolecule assembly

Of the 109,861 super-scaffolds in Aet v3.0 (Extended Data Fig. 1b), 283 (accounting for 95.2% of the total sequence) were anchored on the SNP-based genetic map comprising 7,185 SNP markers^7^ (Extended Data Fig. 1f). The remaining 107,888 super-scaffolds were short and totaled only 199,614,049 bp, accounting for 4.8% of the total 4,224,918,192 bp sequence. Of the 283 large super-scaffolds, the order or orientation of 81 (28.6%) was uncertain (Extended Data Fig. 1f, step 1).

The use of a BNG map for super-scaffolding and pseudomolecule construction is limited by gaps in the map, which are caused by chance clustering of Nt.BspQI nickase restriction sites, making such regions prone to breakage during DNA labelling and electrophoresis. Polymorphism for Nt.BspQI sites can alter the distribution of these fragile regions, and contigs of different BNG maps may therefore overlap. These overlaps could be used to bridge some of the gaps on individual BNG maps, provided that such maps can be aligned. On the basis of this hypothesis, BNG maps were constructed for *Ae. tauschii* acc. CIae23 and *T. aestivum* cv. Chinese Spring (Extended Data Fig. 1d). Contigs of these BNG maps were aligned with the 283 super-scaffolds and AL8/78 BNG contigs (Extended Data Fig. 1g). Polymorphism among the three genotypes did not prevent contig alignments but was sufficient to alter the locations of some of the fragile sites. The use of these two additional BNG maps improved the ordering and orienting of super-scaffolds on the pseudomolecules, leaving only 16 (5.6%) of the 283 super-scaffolds unordered (Extended Data Fig. 1f, step 2). These remaining 16 super-scaffolds were known to be located in the centromeric region on the basis of SNP marker anchoring. However, because the anchoring markers co-segregated, the order and orientation of those super-scaffolds remained unresolved.

To construct pseudomolecules, the gaps between the neighbouring super-scaffolds were filled with 1,000 Ns. Each pseudomolecule started at the tip of the short chromosome arm. The pseudomolecules contained 95.2% of assembled sequences with a total length of 4,025,304,143 bp (Extended Data Fig. 2a). The unanchored super-scaffolds were short and accounted for about 200 Mb. A total of 76 Mb of BNG contigs were devoid of aligned scaffolds. The pseudomolecules and unanchored super-scaffolds comprise assembly Aet v4.0.

### TE annotation

TEs were identified by discovering full-length TEs using structure-based analyses and by scanning the Aet v4.0 assembly with newly identified and previously known TEs to find all (full-length and truncated) elements by RepeatMasker (http://www.repeatmasker.org). A full-length TE is defined as an element with a complete 5′–3′ sequence that includes terminals flanked by target site duplications. For *helitrons*, which have no target site duplications, a 5′-A and 3′-T were required to flank the termini of an element. The structure-based bioinformatic tools used were LTR_FINDER^50^ and LTRharvest^51^ to find LTR-RTs, SINE-Finder^52^ to find SINEs, MITE Hunter^53^ to find miniature inverted-repeat transposable elements (MITEs), and Helitron Scanner^54^ to find *Helitrons*. DNA elements with terminal inverted repeats (TIRs) were identified by integrating a homology search for DDE domains and identification of TIR insertion junctions. The genome was first scanned by BLASTP (*E*-value =10^−10^) using known DDE domains as a query; then, matched regions as well as their flanking sequences were grouped according to the domains they matched and the matched domains were extracted. For each group, closely related subgroups based on the similarity of DDE domains were subsequently identified. For each subgroup, corresponding DNA sequences were aligned and the alignments were inspected for insertion junctions and target site duplications, thus identifying full-length elements. All program outputs were manually inspected to eliminate artefacts. The verified, full-length TEs were then classified into families using previously described criteria^55^. Elements within a family had >80% sequence identity at the DNA level. We constructed an *Ae. tauschii* TE database combining all TE families and used that information to mask the pseudomolecules with RepeatMasker.

To identify new families, representative sequences were compared to known plant TEs in TREP (https://wheat.pw.usda.gov/), PGSB Repeat Element Database (http://pgsb.helmholtz-muenchen.de/plant/recat/), and Repbase (www.girinst.org/repbase/), and families were classified based on their LTRs.

### Age distribution and insertion rate of TE families

Because mutations in LTRs occur at random, two identical LTR-RTs inserted at the same time could have different numbers of mutations. The numbers of mutations on a single LTR with length *l* and inserted *Y* years ago was assumed to follow a Poisson distribution with the rate *rlY*, in which *r* = 1.3 × 10^−8^ mutations per year·per site^56^. The number of mismatches for a given fixed age (time since insertion) on a pair of LTRs is *N*|*Y*, which follows a Poisson distribution with rate 2*rlY.* Negative binomial distributions provided a reasonable approximation of the distributions of mismatches *N* in most of the TE families. By a known probabilistic relation^57^, the age distribution of *Y* follows a gamma distribution. We then used the method of maximum likelihood to estimate the age distributions. The insertion rate *t* years ago was estimated by  where *g*(*t*) was the density of TEs with age *t*, and  was the fraction of surviving elements past age *t*, estimated by fitting an exponential curve to divergence.

### Distribution of TEs along chromosome arms and their ages

To determine whether the distribution of TE families along *Ae. tauschii* chromosomes was homogeneous, mean TE distances to the centromere for the 22 most abundant *Gypsy* and *Copia* families were computed. One-way ANOVA was performed to compare the mean distances of TEs to the centromeres. The global TE family effect was found to be highly significant (*P* < 2 × 10^−16^). Tukey’s test was applied to determine which families were more proximal/distal (Extended Data Fig. 3c).

The hypothesis that LTR-RT families along the centromere–telomere axes of chromosome arms have the same mean ages was tested by separating the 22 families on the basis of their median distance from the centromere into a proximal and distal group, each consisting of 11 families. The difference between the families was statistically tested as described in Extended Data Fig. 3c.

### Gene annotation

Genes were annotated by combining splice site-aware alignments of protein sequences from available public reference datasets with RNA sequencing (RNA-seq) assemblies and inferred putative open-reading frames (ORFs). The ORFs were classified as HCC or LCC on the basis of sequence homology and gene expression support.

### Transcript prediction

The gene annotation pipeline (Extended Data Fig. 4a) combined information of splice site-aware alignments with reference proteins and RNA-seq-based gene structure predictions. Protein sequences from barley (*H. vulgare*)^58^, *B. distachyon*^25^, rice (*O. sativa*)^59^, and sorghum (*S. bicolor*)^18^ as well as predicted ORFs from full-length cDNA sequences from wheat (*T. aestivum*)^60^ were aligned against repeat-masked *Ae. tauschii* pseudomolecules using the splice-aware alignment software GenomeThreader (v.1.6.2; parameters used: -species rice -gcmincoverage 30 -prseedlength 7 -prhdist 4 -force). The resulting transcript structure predictions were then merged using Cuffcompare from the Cufflinks package^61^. The RNA-seq transcriptome data (Extended Data Fig. 4b) contained reads from 24 *Ae. tauschii* samples that were published previously^5^ (Sequence Read Archive, SRA062662, accessions SRR630112 through SRR630135). Additional RNA-seq data (paired end 150-bp reads, quality trimmed to paired end 100-bp reads) were generated by us from *Ae. tauschii* AL8/78 leaf and root tissues from 2-week-old, greenhouse-grown plants, 4-day seedling tissues, developing seeds (10 and 27 days after anthesis) and pooled RNA from developing grains at 10, 15, 20, 27, and 30 days after anthesis. Tri-reagent was used for RNA isolation from leaf and root tissues, and LiCl and acid phenol were used to extract RNA from developing grains^62^.

The RNA-seq reads were aligned against the repeat-masked pseudomolecules using TopHat2^61^ with default parameters. The resulting alignment files were then assembled into transcript structures using the Cufflinks software package^61^. The RNA-seq-based transcript structures were clustered with the reference-based gene model predictions to generate a consensus transcript set using Cuffcompare from the Cufflinks package.

### ORF prediction and selection

A custom script was used to extract transcript sequences on the basis of their coordinates. Transdecoder (version rel_16Jan; parameters: -m 30 –retain_long_orfs 90 –search_pfam pfam.AB.hmm.bin) was applied to determine putative open reading frames as well as the corresponding protein sequences including prediction of PFAM domains. For some transcripts, alternative protein predictions were obtained. All predicted proteins were therefore compared by BLASTP with a comprehensive protein database that contained high confidence protein sequences from *A. thaliana*^63^, *Z. mays*^64^, *B. distachyon*^25^, rice^65^ and sorghum^18^. BLASTP hits with an *E*-value below 10^−5^ were considered significant hits. Sequential filtering was then used to find a single best translation for each transcript. The filtering steps were: (1) with homology support, without homology support but with PFAM domains, and with neither homology support nor PFAM domains; (2) total length of translation; (3) coding sequence (CDS) with start and with stop codon, CDS with start and without stop codon, CDS without start and with stop codon, and CDS without start and without stop codon; (4) number of PFAM domains; and (5) number of significant BLAST hits.

### Confidence assignment

The predicted genes were subjected to stringent confidence classification to discriminate between loci representing HCC protein-coding genes and less reliable LCC genes (Extended Data Fig. 5a), which included gene fragments, putative pseudogenes, and non(-protein)-coding transcripts. Confidence was assigned to a gene in two steps using the criteria and methods described previously^66^. First, genes with transcripts that showed significant homology (BLASTN with *E*-value < 10^−10^) to a repeat element library were considered as low confidence. Second, the predicted peptide sequences were compared with the protein datasets of wheat, barley, *B. distachyon*, rice, sorghum, and *A. thaliana* using BLASTP and hits with homology below 10^−10^ were considered as significant. For each gene, the best-matching reference protein was selected as a template and the transcript sequence with maximum coverage of the template was defined as a gene representative. Genes were defined as HCC if their representative protein had a similarity to the respective template above a threshold (>60% for *A. thaliana*, sorghum, and rice, >65% for *B. distachyon*, and >87% for barley and wheat). This process generated annotation v1.0 and the HCC gene set v1.0 containing 28,847 genes.

To broaden the search for wheat-specific genes and genes with reduced sequence homology but high expression that may have been placed into the LCC class in annotation v1.0, all LCC gene models were re-classified. All predicted protein sequences, including all potential isoforms, were compared with a database containing Triticeae protein sequences from UniProt (downloaded 20 February 2017, filtered for complete sequences) using BLASTP (v.2.60). LCC genes with a significant alignment (*E*-value < 10^−10^, overlap > 95%) to an annotated Triticeae protein were transferred into the HCC class. To include also highly expressed *Ae. tauschii* specific genes, we used Hisat2 (v.2.06) and Stringtie (v.1.3.3) to quantify gene expression in single samples. LCC genes with low sequence homology to reference genomes but strong expression in at least one sample (FPKM > 1) were also considered as additional HCC genes. Finally, 77 LCC genes that were manually identified as RGAs and manually curated gene structures from the prolamin gene family were transferred from LCC into the HCC class. This reclassification and adding additional, manually curated genes produced an annotation v2.0 with updated HCC gene set v2.0 containing 39,622 genes.

Most subsequent gene analyses were performed with both HCC gene sets. As the results were similar, only those generated with the more inclusive HCC gene set v2.0 will be described, unless it is specifically stated that HCC gene set v1.0 was used.

### Validation of annotation

The completeness of gene annotation was evaluated by searching the entire annotation v1.0 with a set of 956 BUSCO genes and BUSCO software (early release, database: plantdb, http://busco.ezlab.org/)^19^. Although 98.4% of the BUSCO genes were correctly annotated, only 90% were correctly assigned to the HCC class. The updated annotation v2.0 was validated with BUSCO v2 (database: embryophyta odb9 containing 1440 BUSCO genes) (Extended Data Fig. 5b).

### Duplicate gene prediction

The program duplicate_gene_classifier from MCScanX^67,68^ was used to detect duplicate genes and classify them as either tandem or dispersed duplicated genes. Tandem duplicated genes were defined as paralogues that were adjacent to each other on the pseudomolecule. All other gene duplications were considered as dispersed, even those in proximity to each other on the same chromosome. The MCScanX parameters were set as follows: Match_score 50, Match_size 5, Gap_penalty -1, overlap_window 5, e_value 1e-05, max gaps = 25. The following genome assemblies were downloaded from Phytozome (https://phytozome.jgi.doe.gov/pz/portal.html) and analysed: *A. thaliana* (TAIR10), *O. sativa* (323_v7.0), and *B. distachyon* (314_v3.1).

### Comparisons with genes annotated in the wheat genomes and other grass genomes

To compare the HCC genes of *Ae. tauschii* with genes annotated in hexaploid bread wheat cv. Chinese Spring (TGACv1 genome assembly and annotation^20^) and other grass genomes, three complementary approaches were used: (1) Best BLAST hit analysis, (2) bidirectional best BLAST hit analysis, and (3) OrthoMCL gene family clustering. All analyses used the HCC gene predictions with one representative gene model for each locus. (1) For the best BLAST hit analysis, all *Ae. tauschii* HCC protein sequences were searched against the TGACv1 wheat HCC gene models (genome-aware) using BLASTP^69^ with an *E*-value cut-off of 10^−5^. Best BLAST hits against TGACv1 wheat gene models were defined by bitscore first, *E*-value second, and percentage identity third. Results for all *Ae. tauschii* gene models with hits are reported in Supplementary Data 2. (2) For the best BLAST hit analysis, both *Ae. tauschii* HCC protein and CDS nucleotide sequences were searched against the TGACv1 wheat HCC gene models using BLASTP and BLASTN with an *E*-value cut-off of 10^−5^ and the TGACv1 wheat HCC gene models were used as queries in searches against the *Ae. tauschii* gene models. (3) Gene family clusters were defined using OrthoMCL version 2.0 (ref. 70). Pairwise sequence similarities between all input protein sequences were calculated using BLASTP with an *E*-value cut-off of 10^−5^. Markov clustering of the resulting similarity matrix was then used to define the orthologue cluster structure, using an inflation value (-I) of 1.5 (OrthoMCL default).

The following datasets were compared: 39,622 *Ae. tauschii* HCC gene models, one representative gene model for each locus; 33,032 gene models of sorghum^18^; 31,694 gene models of *B. distachyon*^25^; 39.049 gene models of rice^71^; 39,734 gene models of barley^72^; and 104,089 protein sequences of high-confidence gene models of Chinese Spring wheat^20^ allocated to the wheat A, B, and D genomes, one representative gene model for each locus. For wheat, there were 32,452 A-genome gene models, 34,712 B-genome gene models, 32,723 D-genome gene models, and 4,202 unknown-genome gene models.

The following statistical comparisons were made with the OrthoMCL gene clusters. The numbers of genome-unique clusters in the wheat genomes were compared with the numbers of genome-unique clusters in the *Ae. tauschii* genome and found to be lower than in the *Ae. tauschii* genome (*P* < 0.0001, two-sided test of proportions, *n* = total numbers of clusters per genome in Fig. 2b). The number of clusters shared by wheat genomes and the *Ae. tauschii* genome was compared with the number of clusters shared by *Ae. tauschii*, barley, *B. distachyon*, rice, and sorghum, and the former was found to be higher than the latter (*P* < 0.0001, two-sided test of proportions, *n* = 15,180, and 12,647). The numbers of B-genome clusters uniquely shared with the D genome and the *Ae. tauschii* genome was compared with those uniquely shared by the A genome with the D genome and the *Ae. tauschii* genome. The B genome shared more clusters with the D genome and *Ae. tauschii* genome than did the A genome (*P* = 0.001 and 0.03 for the D genome and *Ae. tauschii* genome, respectively, two-sided test of proportions, *n* = total numbers of clusters in the A, B, D, and *Ae. tauschii* genomes) (Fig. 2b). Correlation between the number of genome-unique clusters and the number of annotated genes in the genome was also investigated. In the analysis involving the *Ae. tauschii*, barley, rice, *B. distachyon*, and sorghum genomes, *r* = 0.85 (two-tail test, *P* = 0.06, *n* = 5) and in the analysis involving the *Ae. tauschii* and wheat A, B, and D genomes, *r* = 0.98 (two-tail tests, *P* = 0.016, *n* = 4).

In addition to the BLAST analyses in which targets were gene models, a BLAT analysis was performed using wheat nucleotide sequences as targets^20^. CDS nucleotide sequences of 39,622 *Ae. tauschii* HCC genes were used as queries with the BLAT default parameter setting. The best hit of each query was based on the maximum coverage and maximum similarity. Best hits were classified according to the wheat chromosome and genome location of the target sequence.

### Gene distribution along pseudomolecules

To analyse and graph gene density along the centromere–telomere axis of each *Ae. tauschii* chromosome, HCC genes in neighbouring 10-Mb intervals, starting from the tip of the short arm in each pseudomolecule, were counted. A sliding window of gene counts averaging three neighbouring 10-Mb intervals was generated. Two of them overlapped between neighbouring sliding windows. One sliding window was started at the tip of the short arm and the other at the tip of the long arm. The two windows moved in the opposite directions. The corresponding means of the two sliding windows were averaged.

To test homogeneity of the gene density along the chromosomes, we analysed intergenic distances, which have the benefit that gene lengths need not explicitly enter the analysis. A homogeneity assumption on these gene locations corresponds to a homogeneous Poisson process. This has the consequence that intergenic distances should be exponentially distributed. That is, under the assumption of gene distribution homogeneity, the probability density function of intergenic distances is *f*(*x*) = *λ*e^*−λx*^, where *x*, *λ* > 0 and *λ* is the rate parameter. In this setup, the rate parameter *λ* has the interpretation that the expected intergenic distance is 1/*λ*.

Tests for the null hypothesis that the intergenic distances follow an exponential distribution were conducted on an ad hoc basis^27^ using *χ*^2^ goodness-of-fit tests, where some preliminary evidence emerged about the presence of inhomogeneity in gene density. Here we deployed a novel model for intergenic distances using a mixture of exponential distributions, embedding the homogeneous Poisson process into a more general process. A likelihood ratio test was conducted to test the null hypothesis that intergenic distances follow an exponential distribution against the alternative hypothesis that the distances follow a mixture of exponential distributions. This null hypothesis was rejected, indicating that the genes are not uniformly distributed along the chromosomes.

### Prolamin genes

Triticeae prolamin gene sequences including high molecular mass glutenin, low molecular mass glutenin, α-, γ-, ω-, and δ-gliadin genes were used in BLASTN queries against Aet v4.0. Matched sequences with an *E*-value > 10^-10^ were extracted and manually annotated to separate full-length, intact genes from pseudogenes. The coordinates of full-length prolamin genes were included into the HCC gene set v2.0.

### Disease resistance genes

The entire gene set was screened for the presence of RGAs using the RGAugury pipeline^23^. Four classes of RGAs were analysed: NBS-encoding proteins, receptor-like protein kinases, receptor-like proteins, and transmembrane-coiled-coil proteins (Extended Data Fig. 6b). To compute densities of genes in each RGA class along the pseudomolecules (Extended Data Fig. 6c) without the confounding effects of higher gene density in distal chromosome regions, a ratio of the RGAs to the total number of genes was computed using a sliding window of 10 Mb and step length of 8 Mb. That meant that there was 2 Mb of overlapping DNA sequence in consecutive windows along the pseudomolecule (Extended Data Fig. 6c).

A minimum of three RGAs of the same class that were less than 300 kb apart were arbitrarily considered forming a multi-gene locus. A total of 87 such loci were found and their locations are listed in Extended Data Fig. 5d.

### Microsatellites

The microsatellite identification tool MISA (http://pgrc.ipk-gatersleben.de/misa/) was used to discover microsatellite (SSR) motifs from unmasked and masked pseudomolecules and *Ae. tauschii* transcripts. A minimum length of 15 bp was used as a limit. A 1-Mb sliding window was used to calculate the density of genes and SSR motifs, and their correlation was calculated using the R statistical computer package^73^. The Seaborn Python package (https://stanford.edu/~mwaskom/software/seaborn/) was used to produce box plots. A gene density chromosome ideogram was created using the D3js JavaScript library (https://d3js.org/). SSRs were classified on the basis of their length (≥15 bp and ≥20 bp), motif, and location (repeat-masked, repeat-unmasked, and transcripts) (Extended Data Fig. 8a, c, d).

We designed a portal for an SSR search in the *Ae. tauschii* genome sequence (http://aegilops.wheat.ucdavis.edu/ATGSP/data.php, at the ‘SSR search database’ link). The user has a choice to search for SSRs in genes or in intergenic regions, and to use masked or unmasked pseudomolecules. The output gives the characteristics of the SSR, gives the gene name and pseudomolecule coordinates of the gene in or near which it resides. The user can use the name and look up the gene in the HCC gene colinearity database (Supplementary Data 1) or click on the ‘download sequence’ icon and download sequence including the SSR of selected length for primer design or BLAST searches in wheat or *Ae. tauschii*.

### Organellar insertions into the nuclear genome

A BLASTN search was conducted with the *Ae. tauschii* chloroplast genome (GenBank accession, NC_022133) and the *T. aestivum* mitochondrial genome (GenBank accession, NC_00757) sequences against the unmasked and, later, the repeat-masked *Ae. tauschii* pseudomolecules. The top hit was recorded. BLAST hits in the *Ae. tauschii* sequence that were located within 200 bp of one another were merged as single hits. BLAST hits that were encompassed by other BLAST hits were removed. The number of insertions per 10 Mb of DNA were counted and the same sliding window approach as described for genes was used to generate graphs depicting the distribution of organellar insertions across each of the pseudomolecules (Extended Data Fig. 9).

### Locations of telomeric sequences

Sequences of 42 rice telomeric repeats^9^ (TTTAGGG) were concatenated, and unmasked pseudomolecules were BLASTN searched for homology to the sequence. The search output was downloaded and the *E*-value, percentage of identity, and the length of the alignments of detected sequences were recorded. A minimum of five repeats was arbitrarily chosen as a cut-off for considering a hit successful.

### Recombination rate and correlation analysis

The *Ae. tauschii* genetic map containing 7,185 SNP markers^7^ was used as an initial database to compute the recombination rate at each HCC gene. To find correspondence between HCC genes annotated in the pseudomolecules and these SNP markers, the *Ae. tauschii* masked pseudomolecules were searched for homology with the SNP markers using a BLASTN *E*-value < 10^−10^. Genes that were immediate neighbours of SNP markers but were not hit by the BLAST search, received a cM position of a neighbouring marker that was on the map. These empirical data were treated as realizations from functions along the pseudomolecules. First derivatives were estimated from these functions using local polynomial smoothers; these derivatives allowed for computation of the recombination rate at each gene.

Pearson’s correlation coefficients of recombination rate and gene density were computed using the average gene density in a 10-Mb interval as one variable and the recombination rate at the midpoint of the 10-Mb interval as the other.

### Gene colinearity and structural chromosome analyses

Gene colinearity was defined as the shared order of gene starts along two pseudomolecules (one used as a query and the other used as a subject), irrespective of gene orientation. Our arbitrary requirement was that the starts of at least three different genes (five in the study of WGD within the *Ae. tauschii* genome and construction of the Circos) were in an ascending or descending order and that the distances between those genes were <0.5 Mb on the subject pseudomolecule (5 Mb on the *Ae. tauschii* pseudomolecules).

First, a database was created via a homology search of amino acid sequences of the HCC gene set 2.0 on the *Ae. tauschii* pseudomolecules (query) and amino acid sequences of all genes in *B. distachyon*, v3.1, rice, v7.0, and sorghum, v3.1, genome sequences, using BLASTP at an *E*-value of 10^−5^ (subjects). The amino acid sequences of all genes for the four species were downloaded from the Phytozome database. The results were sorted by bit-score in descending order. The top hit for each species was retained as a ‘homologue’ for each *Ae. tauschii* gene for colinearity analyses and comparisons of chromosome structure.

Second, a database was constructed from the first database for each *Ae. tauschii* pseudomolecule with the top hit ordered according to the order of HCC genes along the *Ae. tauschii* pseudomolecule (query) and corresponding hit of subject gene. If an *Ae. tauschii* gene was homologous to tandem duplicated genes on the subject pseudomolecule, only one of the duplicated genes was recorded, provided that it was in a colinear position on the pseudomolecule. Subject gene starts showing an ascending or descending order were recorded. To quantify colinearity, the recorded genes were counted and expressed as a percentage of all genes.

To identify inversions and translocations, the ascending or descending order of genes starts on a subject pseudomolecule was reconstructed by inverting or translocating segments of the pseudomolecules to reconstruct the ancestral colinear gene order. Gene orders in *Ae. tauschii*, *B. distachyon*, rice, and sorghum were compared with each other and ancestral and derived orders were inferred based on maximum parsimony. The data were analysed with a paired *t*-test, using data for individual chromosomes as variables, and Bonferroni correction for multiple comparisons. Smoothed gene-count profiles were generated from the raw counts using local linear smoothers^74,75^.

A database showing colinearity of each of the 38,775 *Ae. tauschii* HCC genes and those along the *B. distachyon*, rice, and sorghum pseudomolecules is in Supplementary Data 1. Cells containing colinear genes are coloured whereas those that are not colinear are colourless. Changes in gene order due to inversions or translocations are indicated by changes in cell colour. For each structural difference, its start and end on the *Ae. tauschii* pseudomolecule is indicated. Also indicated for each change is the branch of the grass phylogenetic tree in which the change had taken place and the type of change. Structural changes are coded as follows: A, inversion of 2 genes; B, inversion of 3 genes; C, inversion of >3 genes; D, translocation of 2 genes within a chromosome; E, translocation of 3 genes within a chromosome; F, translocation >3 genes within a chromosome; iT, intercalated translocation between chromosomes; T, terminal translocation; Dup, duplication of a segment; Del, deletion of a segment.

Inversions and translocations that were discovered in the *Ae. tauschii* genome sequence could be assembly errors, particularly inversions involving only two genes. To evaluate this possibility, the presence of 17 randomly selected two-gene inversions in our independently generated PacBio-Illumina hybrid assembly^6^ of *Ae. tauschii* AL8/78 was determined. All 17 inversions were validated.

### Self-synteny within the *Ae. tauschii* genome

Syntenic blocks within the *Ae. tauschii* genome generated by the pan-grass WGD preceding the divergence of grasses^29^ were identified with the MCScanX package^68^. The ‘all-versus-all’ BLASTP alignments (*E*-value < 10^−5^) was performed. The longest protein was used for each of the 38,775 HCC genes. The alignments were analysed with MCScanX using a maximum gap size of 25 and at least five syntenic genes to define duplicate syntenic blocks. Syntenic blocks within the *Ae. tauschii* genome were drawn using Circos 0.69 software^76^.

### Dot plots

The proteins annotated in *B. distachyon* assembly v3.1, rice v7.0, foxtail millet v2.2, and sorghum v3.1 were downloaded from Phytozome. Only the protein corresponding to the primary transcript was retrieved for each gene. A BLASTP search was conducted of the proteins annotated in *Ae. tauschii* HCC gene set 2.0 against those retrieved for *B. distachyon*, rice, foxtail millet, and sorghum. The top two hits with an *E*-value < 10^−5^ were recorded in separate files. The homologous protein pairs were used to detect syntenic blocks using the software MCscanX^68^ with a match score of 50, gap penalty of −1, *E*-value of 10^−5^, maximum gap size between any two consecutive protein pairs of 25 and a minimum of five consecutive proteins to declare a syntenic region. This was done separately for the top hits (black) and second-best hits (red) (Extended Data Fig. 10d). The MCScanX output was used to draw comparative dot plots.

### Data Availability

The RNA-seq reads were deposited in the European Nucleotide Archive as study PRJEB23317. The pseudomolecules plus the unassigned scaffolds have been deposited into GenBank as Aet v4.0 under BioProject PRJNA341983. All new TE families are listed in Supplementary Data 4. A JBrowser-based genome browser is available at http://aegilops.wheat.ucdavis.edu/jbrowse/index.html?data=Aet%2Fdata%2F&loc. BLAST of pseudomolecules and TEs is available at http://aegilops.wheat.ucdavis.edu/ATGSP/data.php.

## Supplementary information

Life Sciences Reporting Summary (PDF 74 kb)

Supplementary Table 1 This file contains the results of colinearity analysis between the Ae. tauschii pseudomolecules and those of *Brachypodium distachyon, Oryza sativa* (rice), and *Sorghum bicolor* (sorghum). (XLSX 5792 kb)

Supplementary Table 2 This file contains the Best BLASTP results of a comparison between the *Ae. tauschii* genome and those of wheat (the TGACv1 assembly). (XLSX 1571 kb)

Supplementary Table 3 This file contains a list of the 1,762 Resistance Gene Analogues annotated in the *Ae. tauschii* genome sequence. (XLSX 125 kb)

Supplementary Table 4 This file lists all the new TE families discovered in the genome of Aegilops tauschii. (XLSX 2139 kb)

## Data Availability

BioProject
PRJNA341983
European Nucleotide Archive
PRJEB23317

## PowerPoint slides

PowerPoint slide for Fig. 1
PowerPoint slide for Fig. 2
PowerPoint slide for Fig. 3
