# Supplementary material for: Genome sequence of the progenitor of the wheat D genome Aegilops tauschii
Source: Nature. 2017 Nov 1;551(7681):498–502. doi: 10.1038/nature24486 (PMC7416625; doi:10.1038/nature24486)
Supplement: Supplementary file 1 — Life Sciences Reporting Summary (PDF 74 kb) [file 41586_2017_BFnature24486_MOESM1_ESM.pdf]

nature research

Corresponding author(s): Jan Dvorak

☐ Initial submission ☐ Revised version ☒ Final submission

Life Sciences Reporting Summary

Nature Research wishes to improve the reproducibility of the work that we publish. This form is intended for publication with all accepted life science papers and provides structure for consistency and transparency in reporting. Every life science submission will use this form; some list items might not apply to an individual manuscript, but all fields must be completed for clarity.

For further information on the points included in this form, see Reporting Life Sciences Research. For further information on Nature Research policies, including our data availability policy, see Authors & Referees and the Editorial Policy Checklist.

Experimental design

1. Sample size

Describe how sample size was determined.

Fig. 1a: Description of the Ae. tauschii genome. No sampling was performed.
Fig. 1b: All transposon families with >100 complete elements were sampled.
Fig. 1c: An arbitrary sample of plant genomes representative of different genome sizes.
Fig. 1d: and Fig. 1e. Sampling described in Fig.1b above.
Fig. 2a: An arbitrary sample of high-quality grass genome sequences.
Fig. 2b: The number (3) of genomes making up the hexaploid wheat genome.
Fig. 2c: Three genes selected as a minimum for a locus to be considered multigene was arbitrary.
Fig. 3a: The descriptive comparison of gene density and recombination rate. The choice of 10 Mb for the size of the sliding window was arbitrary.
Fig. 3b: No sampling was performed.
Fig. 3c: The choice of 50 genes for the size of the sliding window was arbitrary.
Extended Data Fig. 1 and 2: No sampling was performed.
Extended Data Fig. 3: Sampling described in Fig. 1b above.
Extended Data Fig. 4. No sampling was performed.
Extended Data Fig. 5c: The six genomes were selected arbitrarily. 5d. and 5e: See Fig. 2b above. Fig. 5f: Sample of genomes was arbitrary. Fig. 5g: No sampling was performed.
Extended Data Fig. 6c: Sampling described in Fig. 2c above.
Extended Data Fig. 7a and Fig. 7b: The sizes of the sliding windows (50 genes and 10 Mb) were chosen arbitrarily. Fig. 7c: Subdivision of chromosome into 50 segments was arbitrary.
Extended Data Fig. 8: No sampling was performed.
Extended Data Fig. 9: Sampling described in Extended Data Fig. 7a and Fig. 7b above.
Extended Data Fig. 10a and 10b: The three grass genomes were selected for their high-quality genome assemblies. Fig. 10c: The size of the 50 genes for the non-overlapping window was chosen arbitrarily. Fig. 10e: The boundary between small and large structural changes (>3 genes) was arbitrary.

2. Data exclusions

Describe any data exclusions.

Methods, lines 499-500: Excluded from merging were scaffolds with overlaps <2,000 bp because they could not be reliably merged. Most of them were merged in later steps of the scaffold and super-scaffold assembly.
Methods, lines 512-516: Excluded were reads with quality problems.
Methods, lines 640-644: Some scaffolds could not be included into pseudomolecules primarily because they were too short. They were therefore excluded from estimating the total pseudomolecule length.
Methods, lines 653-658: We failed to anchor some of the super-scaffolds and these were excluded from counting the total number of anchored scaffolds and super-scaffolds.
Methods, lines 769-773: We define here high confidence genes.
Methods, lines 788-790: We excluded reporting data obtained with the HC gene set v1.0 because the results were similar to those obtained with the more inclusive HC gene set v2.0.

Methods, lines 961-963: Excluded were all BLAST hits with lower score value than the top hit because they would likely include paralogues in place of orthologues in the colinearity analysis.
Methods, lines 986-990: We validated only a random sample of two-gene inversions discovered.
Methods, lines 1005-1007: Dot-plots require finding the best match among the duplicated genes that may be present in a genome. Exclusion of predicted proteins not corresponding to the primary transcript was one of the filtering criteria.
Extended Data Fig. 5f, lines 1218-1220. To estimate the numbers of duplicated paralogues, we excluded genes duplicated by the Pan-grass whole genome duplication.
Extended Data Fig. 8d, lines 1278-1281: We describe here the construction of a box-plot and indicate exclusion of extreme values.

3. Replication

Describe whether the experimental findings were reliably reproduced.

We performed two gene annotations in the Ae. tauschii genome and repeated all analyses of the genome with the two gene sets. Our conclusions were essentially identical with both gene sets.

4. Randomization

Describe how samples/organisms/participants were allocated into experimental groups.

Samples were not randomized for the experiments.

5. Blinding

Describe whether the investigators were blinded to group allocation during data collection and/or analysis.

Blinding was not used during data collection.

Note: all studies involving animals and/or human research participants must disclose whether blinding and randomization were used.

6. Statistical parameters

For all figures and tables that use statistical methods, confirm that the following items are present in relevant figure legends (or in the Methods section if additional space is needed).

n/a	Confirmed	
☐	☒	The exact sample size (*n*) for each experimental group/condition, given as a discrete number and unit of measurement (animals, litters, cultures, etc.)
☒	☐	A description of how samples were collected, noting whether measurements were taken from distinct samples or whether the same sample was measured repeatedly
☒	☐	A statement indicating how many times each experiment was replicated
☐	☒	The statistical test(s) used and whether they are one- or two-sided (note: only common tests should be described solely by name; more complex techniques should be described in the Methods section)
☐	☒	A description of any assumptions or corrections, such as an adjustment for multiple comparisons
☐	☒	The test results (e.g. *P* values) given as exact values whenever possible and with confidence intervals noted
☐	☒	A clear description of statistics including central tendency (e.g. median, mean) and variation (e.g. standard deviation, interquartile range)
☒	☐	Clearly defined error bars

See the web collection on statistics for biologists for further resources and guidance.

Software

Policy information about availability of computer code

7. Software

Describe the software used to analyze the data in this study.

All software used is described in Methods

For manuscripts utilizing custom algorithms or software that are central to the paper but not yet described in the published literature, software must be made available to editors and reviewers upon request. We strongly encourage code deposition in a community repository (e.g. GitHub). *Nature Methods* guidance for providing algorithms and software for publication provides further information on this topic.

Materials and reagents

Policy information about availability of materials

8. Materials availability

Indicate whether there are restrictions on availability of unique materials or if these materials are only available for distribution by a for-profit company.	No restrictions

9. Antibodies

Describe the antibodies used and how they were validated for use in the system under study (i.e. assay and species).	Not applicable

10. Eukaryotic cell lines

a. State the source of each eukaryotic cell line used.	Not applicable
b. Describe the method of cell line authentication used.	Not applicable
c. Report whether the cell lines were tested for mycoplasma contamination.	Not applicable
d. If any of the cell lines used are listed in the database of commonly misidentified cell lines maintained by ICLAC, provide a scientific rationale for their use.	*Provide a rationale for the use of commonly misidentified cell lines OR state that no commonly misidentified cell lines were used.*

Animals and human research participants

Policy information about studies involving animals; when reporting animal research, follow the ARRIVE guidelines

11. Description of research animals

Provide details on animals and/or animal-derived materials used in the study.	Not applicable

Policy information about studies involving human research participants

12. Description of human research participants

Describe the covariate-relevant population characteristics of the human research participants.	Not applicable
