# Supplementary figures and images for: Genome sequence of the progenitor of the wheat D genome Aegilops tauschii
Source: Nature. 2017 Nov 1;551(7681):498–502. doi: 10.1038/nature24486 (PMC7416625; doi:10.1038/nature24486)

## Slide 1
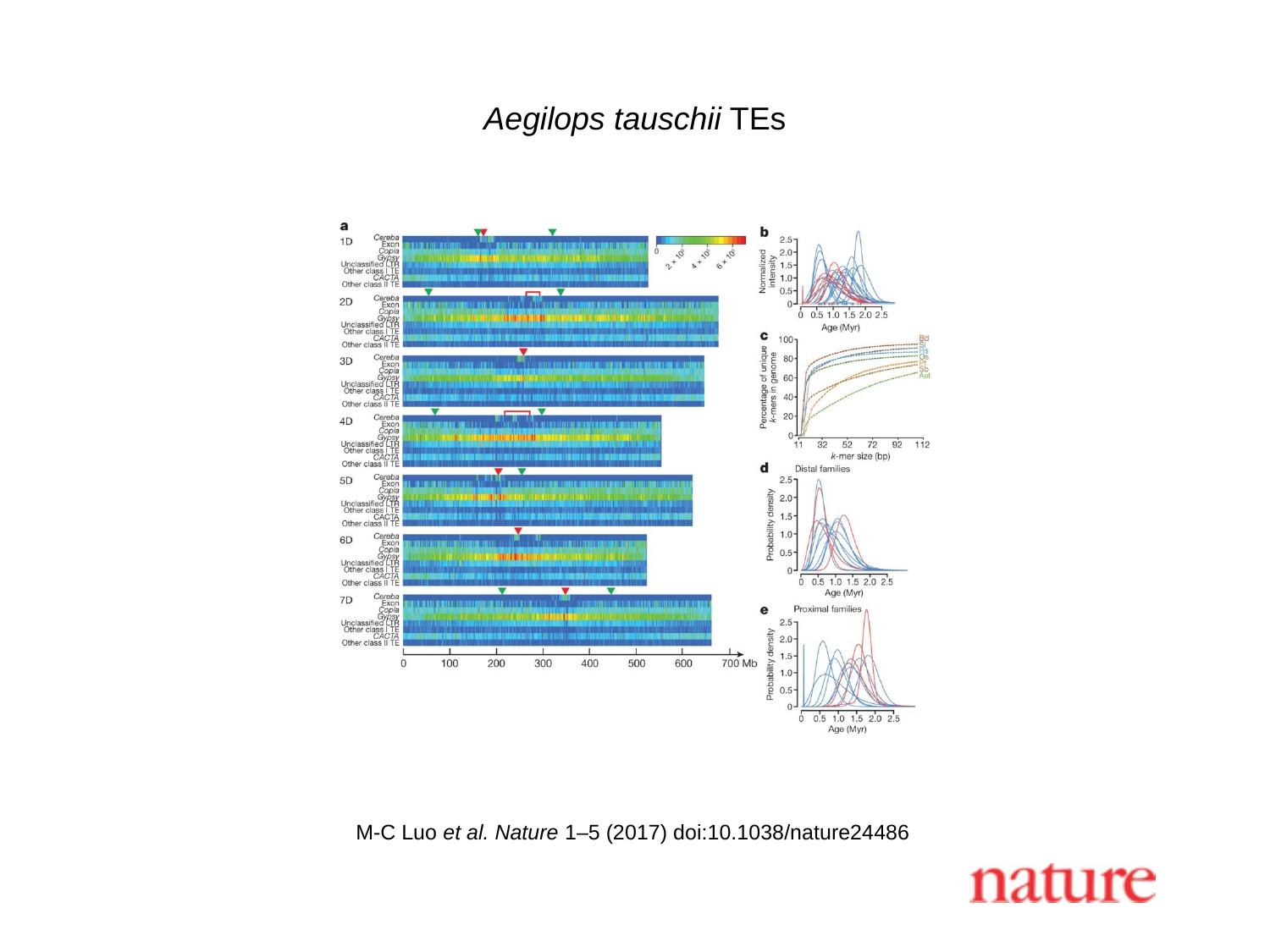

# Aegilops tauschii TEs
M-C Luo et al. Nature 1–5 (2017) doi:10.1038/nature24486

Supplement: Supplementary file 6 — PowerPoint slide for Fig. 1 [file 41586_2017_BFnature24486_MOESM6_ESM.ppt]

## Slide 1
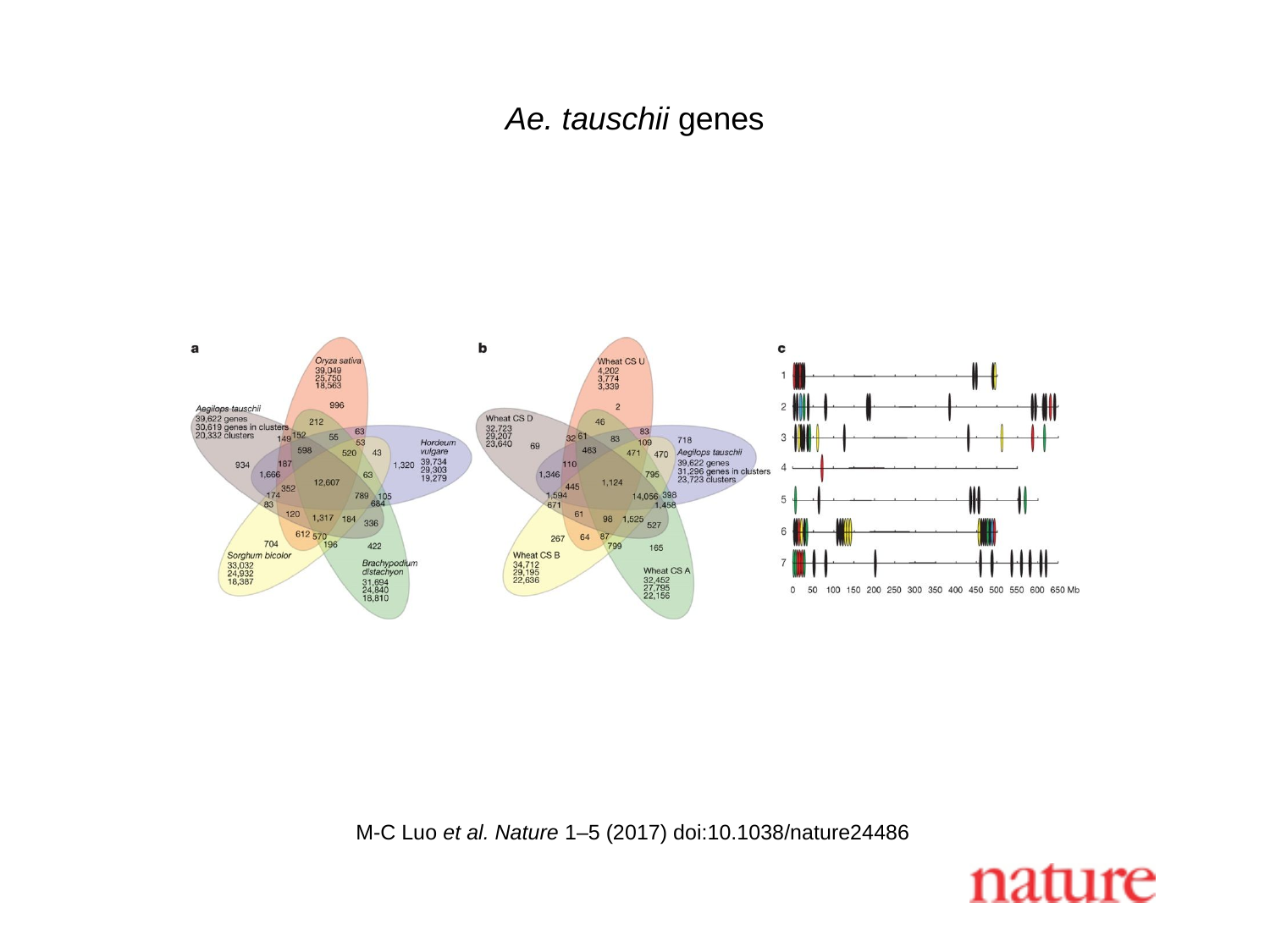

# Ae. tauschii genes
M-C Luo et al. Nature 1–5 (2017) doi:10.1038/nature24486

Supplement: Supplementary file 7 — PowerPoint slide for Fig. 2 [file 41586_2017_BFnature24486_MOESM7_ESM.ppt]

## Slide 1
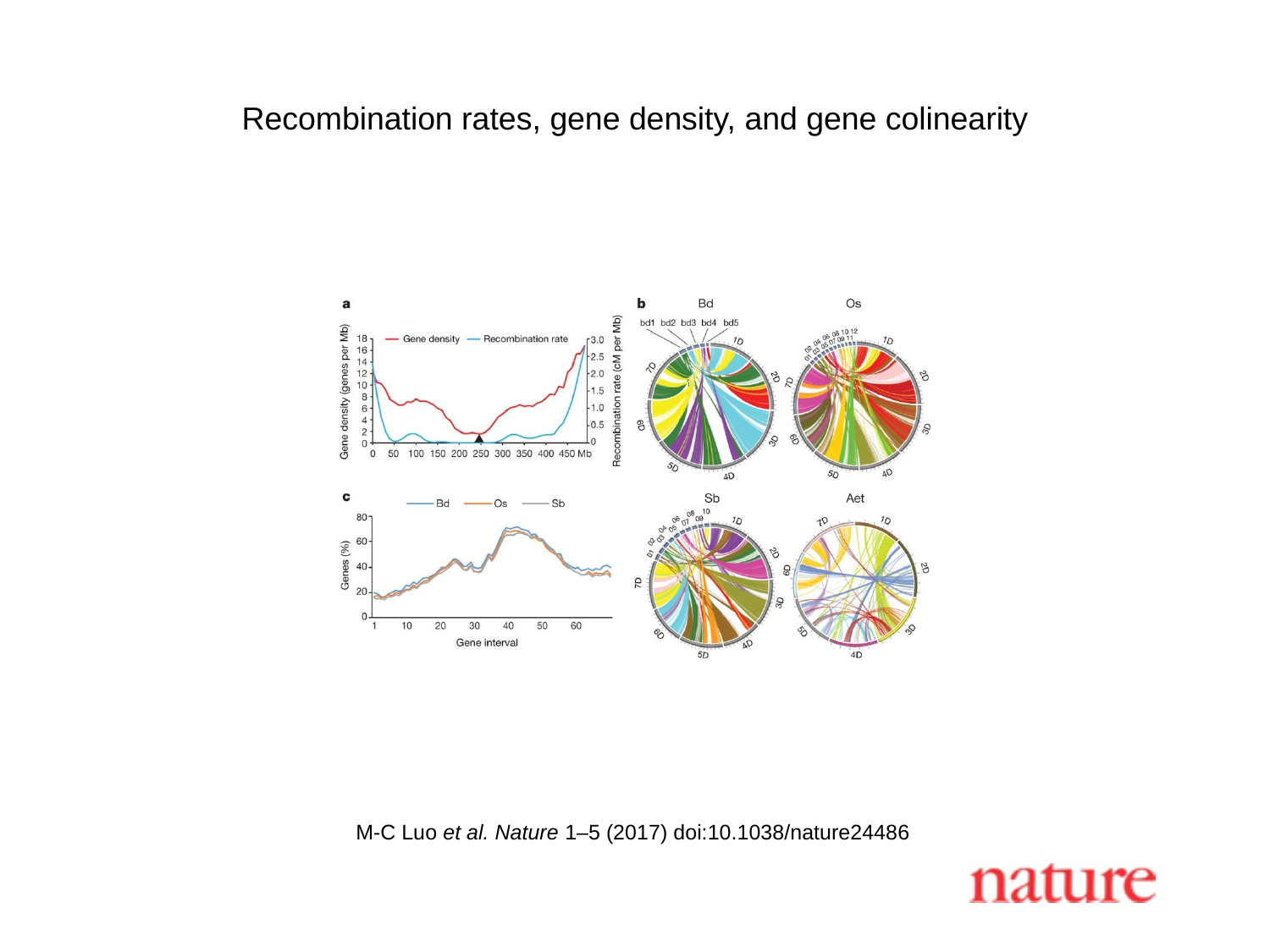

# Recombination rates, gene density, and gene colinearity
M-C Luo et al. Nature 1–5 (2017) doi:10.1038/nature24486

Supplement: Supplementary file 8 — PowerPoint slide for Fig. 3 [file 41586_2017_BFnature24486_MOESM8_ESM.ppt]
